# Bats: Body mass index, forearm mass index, blood glucose levels and *SLC2A2* genes for diabetes

**DOI:** 10.1038/srep29960

**Published:** 2016-07-21

**Authors:** Fanxing Meng, Lei Zhu, Wenjie Huang, David M. Irwin, Shuyi Zhang

**Affiliations:** 1 State Key Laboratory of Estuarine and Coastal Research, East China Normal University, Shanghai 200062, China; 2 Kunming Institute of Zoology, Chinese Academy of Sciences & University of Chinese Academy of Sciences, Kunming 650223, China; 3 Department of Laboratory Medicine and Pathobiology, University of Toronto, Toronto M5S 2E8, Canada; 4 Key Laboratory of Zoonosis of Liaoning Province, College of Animal Science and Veterinary Medicine, Shenyang Agricultural University, Shenyang 110866, China

## Abstract

Bats have an unusually large volume of endocrine tissue, with a large population of beta cells, and an elevated sensitivity to glucose and insulin. This makes them excellent animal models for studying diabetes mellitus. We evaluated bats as models for diabetes in terms of lifestyle and genetic factors. For lifestyle factors, we generated data sets of 149 body mass index (BMI) and 860 forearm mass index (FMI) measurements for different species of bats. Both showed negative inter-species correlations with blood glucose levels in sixteen bats examined. The negative inter-species correlations may reflect adaptation of a small insectivorous ancestor to a larger frugivore. We identified an 11 bp deletion in the proximal promoter of *SLC2A2* that we predicted would disrupt binding sites for the transcription repressor ZNF354C. In frugivorous bats this could explain the relatively high expression of this gene, resulting in a better capacity to absorb glucose and decrease blood glucose levels.

The World Health Organization (WHO) defines diabetes mellitus as a group of metabolic diseases in which blood glucose levels are elevated over a prolonged period of time. Diabetes mellitus is due either to the pancreas not producing enough insulin or the cells of the body not responding properly to insulin^1^. According to WHO, type 2 diabetes, the most common type, represents ~90% of diabetic cases worldwide. Insulin resistance is fundamental to the etiology of type 2 diabetes^2^ because of a combination of lifestyle^3^, including being overweight (a body mass index (BMI) >25) or obese, lack of physical activity, poor diet, stress, and urbanization. Genetic factors, including more than three dozen genes^4^ also may be involved. For type 2 diabetes in humans, obesity is an important lifestyle factor that provides a paradigm for estimating risk factors for health problems^5^.

Bats, order Chiroptera, are the second largest group of mammals. The diets of bats range from fruits, flowers and pollen to insects and other animals as well as blood^6^. This dietary diversity is reflected by different approaches to obtain, store and use energy^7^. The diversity of bats also is reflected by different responses to fasting. Mammals fed protein-rich diets typically show steady plasma glucose levels during fasting. Those fed a carbohydrate-rich diet experience a severe drop in fasting glucose levels^8^,^9^. Bats show different patterns. Pallas’s mastiff bats (*Molossus molossus*) eat insects and maintain constant blood glucose levels through a 48 h fast showing no increase in the concentration of free fatty acids in their blood^10^. Similarly, two new species of New World fruit-eating bats with carbohydrate rich diets maintain relatively high blood glucose levels after a short-term fasts of 2–6 days^11^. These data suggest that frugivorous and insectivorous bats share an ability to maintain glucose homeostasis. Old World fruit-eating bats (e.g., *Rousettus aegyptiacus*) have an unusually large volume (9.1%) of endocrine tissue^12^. New world fruit-eating bats (e.g., *Artibeus lituratus*) have large population of beta cells and elevated sensitivity to glucose and insulin^13^. The Old World great roundleaf bats (*Hipposideros armiger*) generate higher amounts of cold-induced beige fat from white adipose tissue than mice^14^. These data suggest that bats have a good capacity for maintaining glucose homeostasis, lowing blood glucose levels through insulin, and browning the white adipose tissue. Together these features suggest that bats could be good models for understanding lifestyle and genetic factors regulating glucose metabolism, especially for type 2 diabetes.

To aid in diabetes research we have investigated the good properties of sugar metabolism in bats as well as the molecular mechanism underlying them. In Old World Fruit bats, we have previously shown adaptive changes in *SLC2A4*, which encodes glucose transporter 4 and is expressed in adipose and muscles^15^. These changes appear to have improved the ability of bats to respond to high sugar diets^15^. In humans, single nucleotide polymorphisms (SNPs) within *SLC2A2* are associated with the conversion from impaired glucose tolerance (IGT) to type 2 diabetes^16^. Glut2 (encoded by *SLC2A2*), another member of the glucose transport family, functions in the pancreas to mediate glucose uptake for the regulation of insulin secretion and in hepatic cells to allow the generation of hepatic glycogen stores^17^. This could mean that changes in the transcription or translation of *SLC2A2* might affect the abundance of its protein product (Glut2) as well as their efficiency of transporting glucose from blood to the liver during hyperglycemia.

Here, to explore the value of Chiroptera as a model for diabetes in terms of lifestyle and genetic factors, we calculated the body mass index (BMI) and the forearm mass index (FMI) for 149 and 860 species of bats, respectively. We then used both BMI as well as FMI to establish baselines for estimating risk factors for sixteen species of bats with different diets and associated levels of blood glucose. To determine whether fruit-eating bats have better adapted to periodic acute glucose stress than insect-eating bats, we conducted acute glucose tolerance tests on representative species. We then investigated the sequences of the proximal promoters of *SLC2A2* genes as well as the relative abundance of its mRNA product in frugivorous and insectivorous bats to examine the molecular basis for the better capability of fruit-eating bats, as shown by the acute glucose tolerance tests, to lowing blood glucose levels.

## Results

### Body mass index and forearm mass index in bats

Body fat is a risk factor for diabetes in humans and can be estimated using BMI. Standards for normal and overweight bats are lacking. To establish baselines and determine whether BMI, can be used to assess the body mass quantity in bats, we collected a data set of mean weights and sizes of bats from a total of 149 species (see Appendix S1). BMI values were calculated for each of the 149 species of bats, which could be categorized into frugivore, insectivore, and others (sanguivore, carnivore, and omnivore) dietary groups, with sample sizes of 88, 49, and 12 species, respectively. Among these categories, the frugivore group had the highest average BMI, of 7.0 kg/m^2^, with the insectivore group being lowest, with a mean of 3.9 kg/m^2^, and the other bats having an intermediate mean BMI of 5.7 kg/m^2^.

For bats, the number of available forearm length measurements is much greater than the number of full body lengths. Forearm lengths and full body lengths show a positive correlation with each other (R^2^ = 0.933, see Appendix S2), thus we assessed the use of body weight and forearm length to quantify bat body mass. Similar to BMI, FMI is defined as body mass (kg) divided by the square of the forearm length (m^2^). FMI might be a more appropriate measure for body mass quality in bats, as BMI is calculated using total body lengths, which for some, but not all, species include the tail. Thus BMI for species with tails would be lower than that for similar sized bats that lack tails. FMI values were calculated for 860 species of bats, which could be classified as having frugivore, insectivore, carnivore, sanguivore, and omnivore diets and being represented by 244, 581, 8, 3, and 24 species, respectively (see Appendix S1). Similar to BMI, the frugivore group had the highest average FMI value (13.9 kg/m^2^) and the insectivores were lowest (6.1 kg/m^2^), while omnivores (11.6 kg/m^2^) and others (9.6 kg/m^2^) had intermediate FMI values. Both the mean BMI and mean FMI of frugivorous bats are significantly higher than those of the insectivorous bats (*p*-values of 1.021E-013 and 1.263E-098, respectively).

### BMI, FMI and body sizes in bats

**Figure 1:**
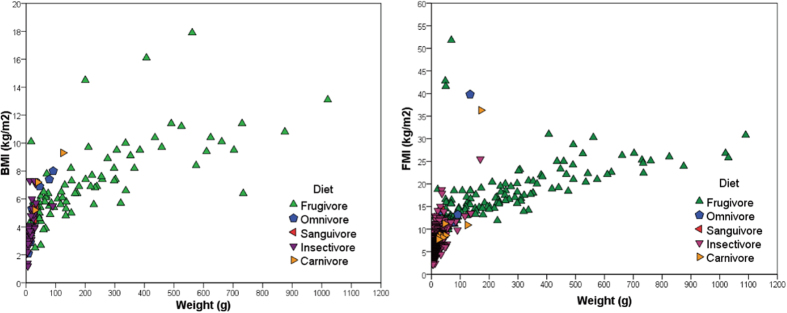
Scatter diagrams of the associations of BMI (left) and FMI (right) to body weight in bats with differing diets. Bats were grouped as frugivorous, omnivorous, sanguivorous, insectivorous, and carnivorous for BMI and indicated by green upward triangles, blue pentagons, red leftward triangles, purple downward triangles, and yellow rightward triangles, respectively.

A scatter diagram of BMI and body weights is used to illustrate the differences in the body sizes of bats with differing diets (Fig. 1A). Among insectivorous bats, 48 of the 49 species group near the origin as they have small body sizes (weight <40 g and BMI <8 kg/m^2^). In contrast, a majority (79.5%, 70 of 88 species) of the frugivorous bats are large, being heavier than 40 g and have BMI values larger than 4 kg/m^2^ (89.8%, 79 of 88). The distribution of FMI and body sizes shows a similar pattern (Fig. 1B). The vast majority of insectivorous bats (85.4%, 496 of 581 species) have a small body size (weight <40 g and FMI <8 kg/m^2^), while only minor proportions of the frugivorous (14.8%, 36 of 244), omnivorous (33.3%, 8 of 24), sanguivorous (0%, 0 of 3), and carnivorous bats (12.5%, 1 of 8) are small. Frugivorous bats represent most bats with body weights heavier than 100 g (93.8%, 91 of 97 species) or have FMI values greater than 15 kg/m^2^ (94.3%, 82 of 87 species). These results show that insectivorous bats tend to have a small body size, while frugivorous bats are larger, and bats with other dietary habits have an intermediate body size. Among frugivorous bats, we also found that Old World fruit-eating bats (OWFBs) have a heavier mean body weight than New World fruit-eating bats (NWFBs) (219.9 vs 20.5 g, *p*-value = 1.558E-043) while NWFBs had higher maximum FMI (10.7 vs 15.6 kg/m^2^, *p*-value = 6.654E-006) (see Appendix S3).

### BMI, FMI and blood glucose in bats

To compare blood glucose levels in bats with differing body sizes, we explored the associations between BMI and FMI with blood glucose levels. We obtained blood glucose level, BMI, and FMI data for a total of sixteen species of bats. Data for *Scotophilus heathi*^18^, *Epomophorus wahlbergi*^19^, *Pteropus vampyrus*^20^, *P. hypomelanus*^20^, *Rousettus aegyptiacus*^20^, *Artibeus intermedius*^21^, *Taphozous nudiventris*^22^ and *Nyctus noctula*^23^ were retrieved from published papers, with the means data, and their upper and lower limits, used for calculating BMI and FMI. Data for the remaining species of bats (i.e., *Myotis daubentonii*, *M. ricketti*, *Rhinolophus affinis*, *R. sinicus*, *R. ferrumequinu*, *Rousettus leschenaulti*, *Hipposideros armiger*, and *H. pratti*) were obtained in this work (see Appendix S4).

**Figure 2:**
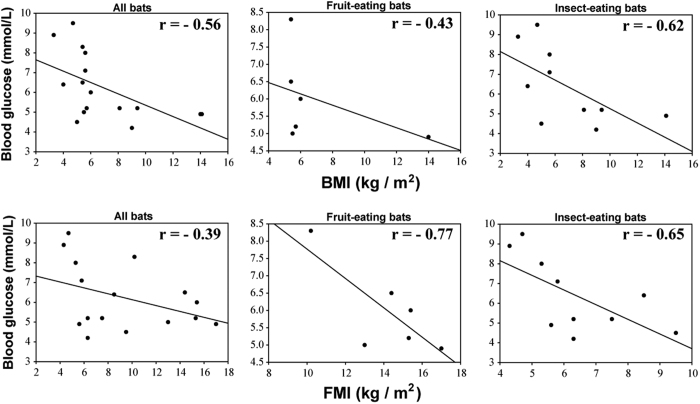
Scatter diagrams of the associations of blood glucose to BMI (above) and FMI (below), and linear regression curves for sixteen bats as a single group or divided into two dietary groups. BMI and FMI, and blood glucose concentrations are shown as kg/m^2^ and mmol/L, respectively. The correlation coefficients (r) were shown above the linear regression analysis curves.

When the sixteen bats are considered as single or as two dietary (insectivore and frugivore) groups, both BMI and FMI show an inter-species negative correlation with blood glucose levels (Fig. 2). Among the frugivores, *P. vampyrus* has the highest BMI (14.0 kg/m^2^) and FMI (17.0 kg/m^2^) but the lowest blood glucose level (4.9 mmol/L), while *C. sphinx* has the lowest BMI (5.4 kg/m^2^) and FMI (10.2 kg/m^2^) and highest blood glucose level (8.3 mmol/L). For insectivores, *T. nudiventris* has the highest BMI (14.1 kg/m^2^) and third lowest blood glucose level (4.9 mmol/L), with *R. affinis* having the highest blood glucose level (9.5 mmol/L) and the second lowest FMI (4.7 kg/m^2^). At the inter-species level, bats with larger body sizes tend to have lower blood glucose levels. In contrast, a positive correlation between body size and blood glucose level is usually found in human (intra-species) comparisons^24^.

### Response of fruit-eating and insect-eating bats to an intraperitoneal glucose tolerance test

**Figure 3:**
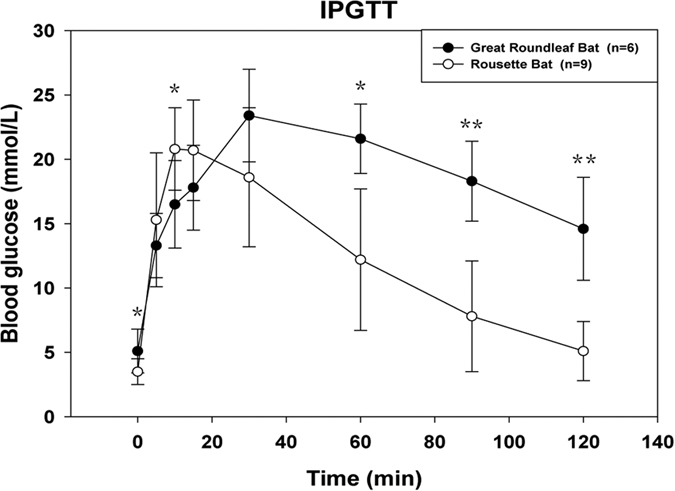
IPGTT results for the Rousette and great roundleaf bats. Nine frugivorous Rousette bats (*Rousettus leschenaultii*) and six insectivorous great roundleaf bats (*Hipposideros armiger*) were used for intraperitoneal glucose tolerance test (IPGTT). Bats were acclimatized for one month prior to the IPGTT. Bats were intraperitoneally injected with volumes of glucose (100 mg/ml) corresponding to their body weight to yield 2 g of glucose per kilogram body mass. Blood samples were collected out to 120 min. Data are shown as mean ± SD. Blood glucose levels of the Rousette bats and great roundleaf bats are shown as empty and black dots, respectively. Significant differences were assessed using two-tailed Student’ *t* tests between the bats groups and significance is indicated by one (P < 0.05) or two (P < 0.01) asterisk.

Species with a capacity to rapidly decrease blood glucose levels are good models for diabetes research. To examine whether, and to what extent, bats have adapted to periodic acute glucose stresses, we conducted intraperitoneal glucose tolerance tests (IPGTT) on nine individuals of a representative fruit-eating bat (*R. leschenaultii*) and six individuals of a representative insect-eating bat (*H. armiger*). Blood glucose levels at the resting state in both *R. leschenaultii* and *H. armiger* are low, but rapidly increase after an intraperitoneal injection of glucose, peaking within 30 min, and then steadily decline until the last time point of our test (Fig. 3). Significant differences in blood glucose levels (*p*-values range from 0.000 to 0.038) between the two bat species were observed at 0, 10, 60, 90, and 120 min after glucose injection (Fig. 3). The area under the curve (AUC) for the blood glucose test in *R. leschenaultii* and *H. armiger* were 24.9 h · mmol/L and 38.0 h · mmol/L, respectively, with the AUC for *H. armiger* being 52.6% greater than that for *R. leschenaultii*, indicating that the frugivorous bat has a better capacity to absorb glucose and decrease blood glucose level compared to the insectivorous bat.

### Proximal promoter sequence of *SLC2A2* in bats

As Glut2 (encoded by *SLC2A2*) is involved in transporting glucose into the liver when blood glucose level rise, it is reasonable to assume that elevated transcriptional or translational levels of *SLC2A2* gene should lead to a more rapid decrease in blood glucose level through the transport of glucose from blood into the liver by Glut2. To assess whether changes in Glut2 abundance may have occurred we examined the proximal promoter sequence of *SLC2A2* as well as mRNA abundance in bats.

**Figure 4:**
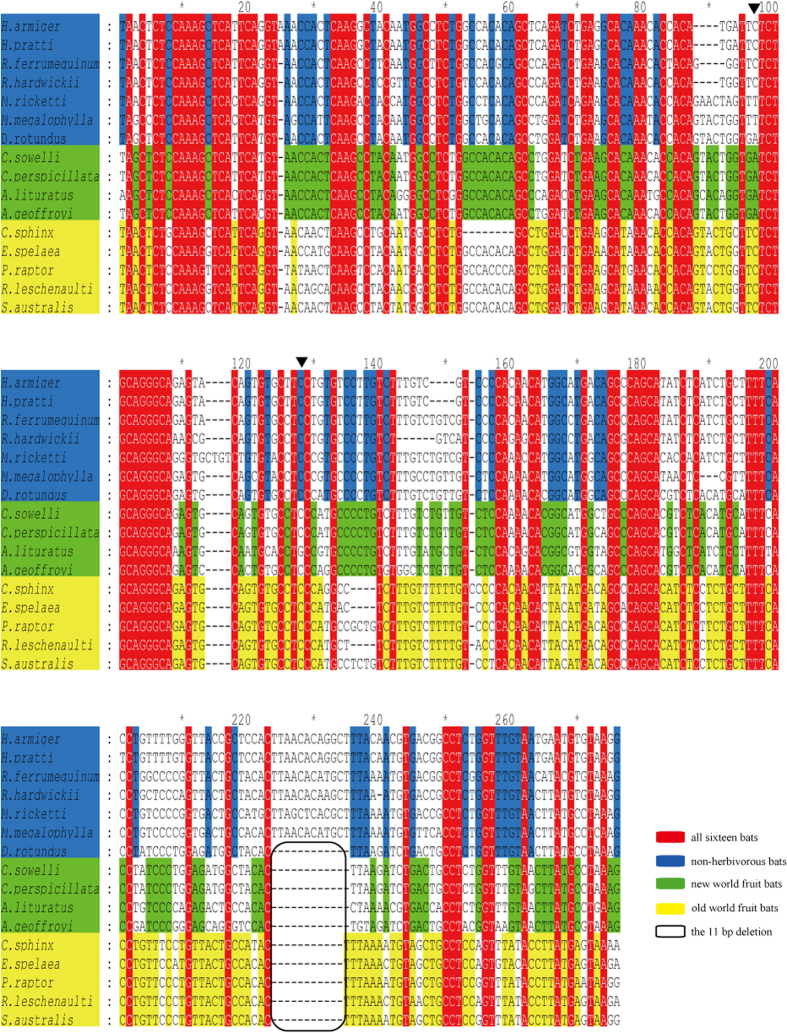
Aligned partial proximal *SLC2A2* promoter sequences from sixteen species of bats. Bats were divided into three groups, namely New World fruit-eating bats (NWFBs), Old World fruit-eating bats (OWFBs), and non-frugivorous bats. Conserved sites in each of the three groups, together with the sequence names, are shadowed in green, yellow, and blue, respectively. Completely conserved sites in all sixteen bats are shadowed in red. The 11 bp deletion is in a black box. Base numbers are shown above the sequences. Bases homologous to human promoter SNPs rs5393 and rs5394 of *SLC2A2* gene are at positions 97 and 124 of the aligned sequences and are indicated by the downward black triangles.

Partial *SLC2A2* proximal promoter sequences of four NWFBs, five OWFBs, one blood-eating bat, and six insect-eating bats were amplified using two pairs of primers (accession numbers KT961103 to KT961117 and KU162944, see Table 1). The sizes of the sequences obtained from the sixteen bat species ranged from 248 to 267 base pair (bp) and their alignment (275 bp) is shown in Fig. 4. We divided the bat species into three groups, NWFBs, OWFBs, and non-frugivorous bats, based on their diets. Among the 275 aligned sites in the promoter sequence, 79 (shadowed in red) are perfectly conserved within the 16 bat species examined. Within NWFBs, and OWFBs, and non-frugivorous bats, 114 (labeled in green), 125 (yellow), and 86 (blue) additional sites are perfectly conserved, respectively (see Fig. 4), yielding levels of conservation of 70.2%, 74.2%, and 60.0%, respectively, for these three dietary groups, showing the strong purifying selection pressure acting in frugivorous bats.

### Changes in the rates of evolution of the *SLC2A2* proximal promoter

**Figure 5:**
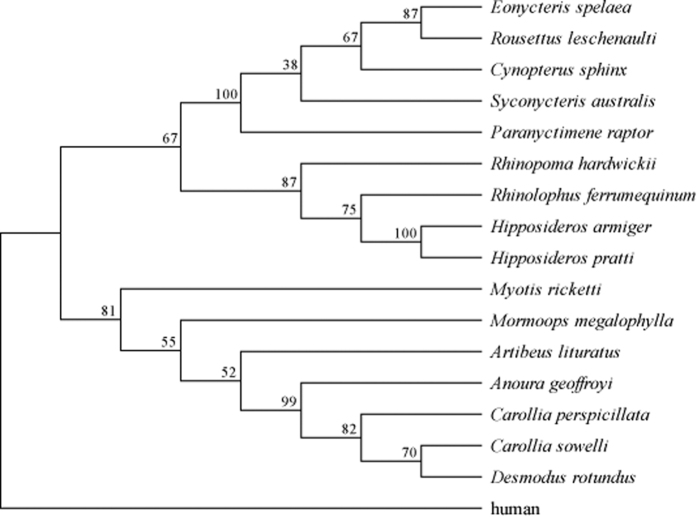
Reconstructed maximum likelihood (ML) phylogeny of the partial *SLC2A2* promoter sequences from sixteen bats with human as the outgroup. Bootstrap (BS) values are shown for each node.

Changes in promoter sequences can result in differences in the binding patterns of transcription factors. The gain and/or loss of transcription factor binding sites should lead to changes in the evolutionary constraints acting upon the sequence, resulting in differences in evolutionary rates in different species lineages and it can be detected by relative rate test^25^. As a first step to examine the relative rates of evolution in the *SLC2A2* proximal promoter, we reconstructed the phylogeny of the promoter sequences using maximum likelihood (Fig. 5). The reconstructed phylogeny resolved all of the species and is largely consistent with the accepted species tree for bats, except for the grouping of the common vampire (*Desmodus rotundus*) with the Sowell’s short-tailed bat (*Carollia sowelli*). We used both the reconstructed phylogeny and the accepted bat species tree for all of the following molecular analysis, with both trees generating consistent results that only slightly differed with respect to *p*-values. The following are the results generated using the accepted bat species tree. When the accepted species tree was used, the null hypothesis of an equal evolutionary rate for the *SLC2A2* promoter sequence for all lineages among bats was rejected at the 5% significance level (*p-*value <0.01), indicating that differences in the evolutionary rates for *SLC2A2* promoter sequences exists between lineages. When non-frugivorous bats were examined, no differences among the species were detected in the relative rate test. Among the four NWFBs, the Geoffroy’s tailless bat (*Anoura geoffroyi*) showed a different rate of evolution in all pairwise comparisons with other NWFBs, while among the five OWFBs, the southern blossom bat (*Syconycteris australis*) showed a significantly difference in its evolutionary rate compared to the lesser dawn bat (*Eonycteris spelaea*) (*p-*value = 0.012), but only had marginal *p-*values in comparisons with Leschenault’s rousette (*Rousettus leschenaulti*) (0.052), greater short-nosed fruit bat (*Cynopterus sphinx*) (0.052), and unstriped tube-nosed bat (*Paranyctimene raptor*) (0.058). No significant differences in bats were detected among the remaining four OWFBs (*p-*values ranging from 0.371 to 1.000). These results show that some, but not all, fruit-eating bats have experienced changes in the rate of evolution for the proximal promoter sequence of *SLC2A2*.

### Predicted transcription factor binding sites of bats *SLC2A2* promoter

To investigate whether the changes in rates of evolution of the proximal promoter for *SLC2A2* in fruit-eating bats is associated with changes in potential transcription factor binding sites, and thus possibly the constraints acting upon the sequences, we predicted putative transcription factor binding sites in these sequences using JASPAR (http://jaspar.genereg.net)^26^. When a 99% relative profile score threshold was used, the five most frequently predicted binding sites were those for SOX10, Nkx2-5(var.2), MZF1, MEIS1, and MAFG::NFE2L1, which were identified in 13, 9, 8, 8, and 7 of the 16 proximal promoter sequences, respectively (see Table 2). When diets of the bats was considered, putative SOX10 binding sites were identified in proximal promoters of *SLC2A2* of species in all three groups (NWFBs, OWFBs, and non-frugivorous bats), while putative Nkx2-5(var.2) sites were found in NWFBs and non-frugivorous bats, MEIS1, MZF1, and MAFG::NFE2L1 sites in OWFBs and non-frugivorous bats, ZNF354C sites in non-frugivorous bats, and a FOXL1 site in OWFBs. These results show that potential changes in the regulation could exist between different species of bats. Intriguingly, a binding site for ZNF354C was found only in non-frugivorous bats.

Further examination of the partial *SLC2A2* promoter sequences identified an 11 bp deletion in NWFBs and OWFBs, starting from position 227 of the alignment (see Fig. 4). This deletion, in fruit-eating bats, overlaps a portion of the human *SLC2A2* promoter sequence that is predicted to bind ZNF354C, a transcriptional repressor^27^. Loss of this ZNF354C binding site in fruit-eating bats suggests the possibility that these bats might have increased transcription of *SLC2A2*.

### Relative quantitative real-time PCR for bats

To determine whether the deletion in the *SLC2A2* proximal promoter found in frugivorous bats is associated with differences in gene expression, we conducted quantitative real-time reverse transcriptase PCR (qRT-PCR) for *SLC2A2* mRNA using RNA isolated from the livers two species of frugivorous (with the 11 bp deletion) and two species of insectivorous (without the 11 bp deletion) bats. Primers for qRT-PCR were designed to the conserved region near the 3′ end of the *SLC2A2* coding sequence identified in bats (see Primer design Section below). Among the four bat species examined, an insectivorous bat (*M. ricketti*) had the lowest mean *SLC2A2* mRNA expression level, which was arbitrarily defined as one. The abundance of *SLC2A2* mRNA in the other insectivorous bat (*H. armiger*) was 1.3 times higher, however, the two frugivorous bats had much higher relative expression levels, of 7.2 times higher for *C. sphinx* and 31.0 times higher for *R. Leschenaulti*. These results show that the mRNA abundance for *SLC2A2*, and potentially its product Glut2, is much higher in the liver of frugivorous bats. Preliminary western blot analysis of proteins from the livers of these bats show similar patterns (results not shown).

## Discussion

Here we have evaluated the bat as a potential model for studying diabetes in terms of its lifestyle and genetic factors. Since obesity is an important lifestyle factor for diabetes in humans, we investigated whether body mass index (BMI) might be a suitable index and representative of blood glucose levels in bats. However, the presence of tails within bats is variable, thus similar sized bats may have very different full body length due to the presence or absence of a tail. This suggests that’s BMI might not be a useful proxy for body mass. We considered the possibility that the length of the forearm, which supports the wing necessary for flight, might be a better proxy for calculating body mass index, which we call FMI (forearm mass index). Lengths of the forearm for large number of bat species exit, and outnumber the number of species with known full body lengths. Forearm length and full body length show a positive correlation with each other (R^2^ = 0.933, see Appendix S2), indicating that forearm length should be useful for evaluating mass content in bats. We generated FMI and BMI values for 860 and 149 species of bats, which range from 2.2 ~ 51.8 kg/m^2^ and 1.2 ~ 17.9 kg/m^2^, respectively. Compared with BMI values seen in human BMI (normal healthy individuals have BMI = 19 ~ 25 kg/m^2^), both BMI and FMI in healthy bats are much lower. BMI and FMI standard values for normal and overweight bats will need to be determined for each species of bat.

Here we used BMI and FMI to explore baseline body mass quantity for bats with differing diets. Both BMI and FMI were consistently higher in frugivorous bats (fed on fruits, nectar, and pollen) than in insectivorous bats, which had the lowest average BMI and FMI values, while omnivores (who fed on both fruit and insects or vertebrates) and other bat (fed on blood or vertebrates) species have intermediate BMI and FMI values. These differences in values might reflect the evolutionary history of body size in bats, from small insectivorous ancestor (with low body weight and low BMI, FMI) to partially or exclusively frugivorous species with larger or heavier bodies (high body weight and high BMI, FMI). For humans, higher BMI values often indicate increased blood glucose levels and an increased risk for diabetes^24^. To our knowledge BMI has not been used to assess diabetic risk in any other species. Here we show that both BMI and FMI of diverse bat species show a negative correlation with blood glucose levels (see Fig. 2). This was especially obvious for the frugivorous species, suggesting that frugivorous bats, those bat species with lower baseline body weights (lower BMI or FMI) have higher blood glucose levels, while those with relatively higher baseline body weights have lower blood glucose levels. Lower baseline body weight, however, does not necessarily mean greater resistance to elevated blood glucose levels causing gain of body weight. The negative associations between both BMI and FMI with blood glucose levels in frugivorous bats likely resulted from an adaptation to a fruit-eating diet. Bats are the only mammals capable of true self-powered flight, which is a mode of motion that consumes a high amount of energy. Mammals fed protein-rich diets show resistance to fasting compared to those fed a carbohydrate-rich diet^8^,^9^. The energy gap, between energy supplied from food and energy expenditure for flight, is a threat that bats face daily. We hypothesize that as a response to strong evolutionary pressure to close the energy gap, frugivorous bats have elevated their blood glucose levels, such that a large amount of energy is readily available for flight, despite eating relatively low energy foods compared to insectivorous bats. Additional studies into the associations of blood glucose levels and their FMI values in bats, and the monitoring of blood glucose in omnivorous bats fed protein- or carbohydrate-rich diets should provide a better understanding of the mechanisms used to maintain glucose homeostasis.

To determine whether, and to what extent, fruit-eating bats have adapted to the periodic acute glucose stresses they experience, in comparison to insect-eating bats, we examined the responses of bats with different dietary habits to an intraperitoneal glucose tolerance test (IPGTT). The frugivorous Rousette bats showed a stronger ability at lowering their blood glucose levels when challenged to maintain glucose homeostasis. Examination of pancreatic tissue sections show a greater number and larger mass of beta cells in Rousette bats compared with the great roundleaf bat (data not shown), which is similar to results observed in another OWFB, *Rousettus aegyptiacus*, which contained an unusually large volume (9.1%) of endocrine tissue^12^. A NWFB, *Artibeus lituratus*, has been shown to contain a large population of beta cells and have an elevated sensitivity to glucose and insulin^13^. Additional frugivorous bats, especially NWFBs, should be investigated by IPGTT and other glucose tolerance tests.

Since Glut2 (encoded by *SLC2A2*) is involved in the lowering of blood glucose levels during hyperglycemia, through transporting glucose from blood into liver, it is reasonable to assume that elevated transcription or translation of this gene will lead to a more rapid decrease in blood glucose level. Therefore we examined the transcription of *SLC2A2* mRNA in the livers of bats. To address this, we amplified part of the proximal promoter for *SLC2A2* from 16 species of bats yielding an alignment of 275 bp (Fig. 4). The amplified sequences represent bats from six families of the two main suborders of bats and include non-frugivorous, NWFBs, and OWFBs. OWFBs and NWFBs showed higher conservation of the nucleotide sites within the amplified promoter sequences, indicating that strong purifying selective pressure acts within frugivorous bats. As a bidirectional glucose transporter^17^, Glut2 shuttles glucose between blood and cells in the liver, as well as other tissues, and thus allows frugivorous bats to quickly import glucose into the liver after a meal and to export glucose when fasting.

To determine whether changes in the rate of sequence evolution of the partial promoter sequences had occurred among bats with different dietary habits, we applied Tajima’s relative rate tests^25^ using the sequences from the NWFBs, OWFBs, and non-frugivorous bats. Evidence for unequal rates were only detected for the Geoffroy’s tailless bat and the southern blossom bat. The Geoffroy’s tailless bat feed on nectar, fruit and pollen, but may also visit flowers to obtain insects^28^. The southern blossom bat is nectarivores and feed exclusively on pollen and nectar^29^. The occasional insect-eating habit of the Geoffroy’s tailless bat and the exclusively pollen- and nectar-eating habit of the southern blossom bat might drive changes in the rates of evolution of the *SLC2A2* proximal promoter in these species. Among frugivorous bats, NWFBs displayed higher levels of between- and within-group disparities than OWFBs, possibly due to their greater diversity in feeding habits, which is unparalleled by any other family of mammals^30^. In addition to the high similarities in their promoter sequences, all nine fruit bats examined in this study shared an 11 base deletion in the proximal *SLC2A2* promoter. This deletion causes the loss of a predicted binding site for the transcriptional repressor ZNF354C^27^, which might allow higher expression of *SLC2A2* in fruit bats. Results from both qRT-PCR and western blot experiments of liver tissue demonstrate higher transcriptional and translation abundance of *SLC2A2* (and Glut2) in frugivorous bats than insectivorous bats, which likely results in a better capacity to absorb glucose and maintain glucose homeostasis in frugivorous bats.

Our cloned bat *SLC2A2* promoter sequences also covered two sites homologous to promoter SNPs associated with diabetes in humans^16^ (see Table 3). For the position homologous to the human rs5394, all sixteen bats examined in this study, except one NWFB, shared the human susceptible SNP allele, while for the site homologous to SNP rs5393, the common vampire bat (*D. rotundus*) as well as all four NWFBs, but not the five OWFBs, shared the human susceptible allele (see Table 3). Two species of NWFBs have previously shown to maintain relatively high blood glucose levels after a short-term fast (2–6 days)^11^, suggesting that these NWFBs evolved mechanisms to adapting to their carbohydrate-rich diets. We hypothesize that the high percentage of shared bases in NWFBs homologous to diabetes susceptible alleles in humans is not the result of random changes but instead is the result of natural selection for maintaining sequences associated with elevated blood glucose levels in NWFBs. The common vampire bat exhibits an unusual susceptibility to starvation, and their blood glucose levels are reduced to remarkably low levels for vertebrate survival after a 24 hour fast^31^. These sanguivorous bats have a food sharing behavior that allows the recipients to survive for at least an additional half day before starvation^32^,^33^. Thus the sharing of the base homologous to the human *SLC2A2* rs5393 susceptible allele may provide the vampire bat with an advantage in increasing their blood glucose levels during starvation. Additional investigations into genes underlying glucose absorption and the decrease in blood glucose levels in bats, coupled with studies into the association of bats blood glucose levels and their FMI values and the monitoring of blood glucose levels in omnivorous bats fed protein- or carbohydrate-rich diets, should provide a better understanding of the mechanisms bats use to maintain glucose homeostasis.

## Materials and Methods

### Ethics Statement

All experiments with bats described in this study were carried out in accordance with approved guidelines and were approved by the East China Normal University Animal Welfare Committee (Ethic certificate no. AR2012/03001).

### Measurement and statistical analysis

Data on bat body weights, full body lengths, and forearm lengths for bats were collected in field experiments or were retrieved from the online Encyclopedia of Life database (http://eol.org/)^34^. Full body and forearm lengths for the bat were defined as the distance along the longest body axis from head to tail and from elbow to wrist, respectively. Body and forearm mass indexes, with unit as kg/m^2^, were defined as the body weight divided by the square of full body or forearm lengths, respectively. Bats were divided into frugivore, insectivore, carnivore, omnivore, and sanguivore groups based on their diets. A single factor analysis of variance (ANOVA) and Tamhane post hoc tests were used to determine which dietary group(s) was significantly different from the others.

### Intraperitoneal glucose tolerance test

To examine the response of bats to an acute glucose stress, nine fruit-eating bats (*Rousettus leschenaultia*) and six insect-eating bats (*Hipposideros armiger*) were examined by intraperitoneal glucose tolerance tests (IPGTT). The average body weights (mean ± S.D.) for *R. leschenaultii* and *H. armiger* were 81.2 ± 7.2 g and 48.4 ± 7.2 g, respectively. All bats were acclimatized to the laboratory for one month prior to conducting the IPGTT. Bats were intraperitoneally injected with a volume of glucose (concentration 100 mg/ml) that corresponds to 2 g of glucose per kilogram body mass of the individual. Blood glucose concentrations were measured using an ACCU-CHEK Integra (Roche, Swiss) at 0, 5, 10, 15, 30, 60, 90, and 120 min after the glucose injection from a drop of blood sampled from the forearm. Levene’s test was used to assess the homogeneity of the variance of the blood glucose concentrations between the frugivorous and insectivorous bat groups. Student’s *t*-test was used to determine if the blood glucose concentrations between the fruit-eating and insect-eating bats at the different time points were significantly different from each other.

### Species list

A total of sixteen bats, from six families, were used for the cloning of *SLC2A2* promoter sequences. The sampled species included eight species from both Yinpterochiroptera and Yangochiroptera (see Table 1). The sampled bats were five OWFBs (*Cynopterus sphinx*, *Rousettus leschenaultia*, *Eonycteris spelaea*, *Paranyctimene raptor*, and *Syconycteris australis*), four NWFBs (*Anoura geoffroyi*, *Carollia perspicillata*, *C. sowelli*, and *Artibeus lituratus*), six insectivorous bats (*Hipposideros armiger*, *H. pratti*, *Rhinolophus ferrumequinum*, *Myotis ricketti*, *Rhinopoma hardwickii*, and *Mormoops megalophylla*), and one sanguivorous bat (*Desmodus rotundus*). For quantitative real-time reverse transcriptase PCR (qRT-PCR), two fruit-eating bats (*C. sphinx* and *R. leschenaultia*) and two insect-eating bats (*H. armiger*, and *M. ricketti*) were used. For qRT-PCR, two individuals from each species, one male and one female, were used. qRT-PCR was conducted twice for each individual.

### Nucleic acid Extraction

Genomic DNA was extracted from bat wing membranes or muscle specimens using TIANamp Genomic DNA Kit (Tiangen, China). Total RNA from the liver was extracted with the RNAiso reagent (Takara, Japan) according to the manufacturer’s protocol and treated with RQ1 RNase-Free DNase (Promega, USA) to remove contaminating DNA. Concentrations of the genomic DNA and total RNA were measured by a spectrophotometer SMA4000 (Merinton, USA). Total RNA (5 μg) was reverse-transcribed into cDNA using High-Capacity cDNA Reverse Transcription Kits (Applied Biosystems, USA) and stored at −20 °C until use.

### Primer design

*SLC2A2* gene sequence data from two species of bats, *Pteropus vampyrus* and *Myotis lucifugus*, were retrieved from the Ensembl database and used to design primer for the partial cloning of the *SLC2A2* proximal promoter region in bats. To design primers for qRT-PCR, we first designed a pair of primers (Glut2-CDS-F and -R, see Table 4) that was used to clone part of the *SLC2A2* coding sequence. These sense and anti-sense primers were designed to different exons to eliminate the potential interference by genomic DNA. Next, based on the alignment of the partial *SLC2A2* coding sequences, we designed primers within conserved sequences for qRT-PCR (Glut2-Q-F and -R, see Table 4). Products amplified by the pair of qRT-PCR primers were sequenced to confirm that amplification of the correct coding sequences occurred in all bat species. The amplification efficiency of the qRT-PCR primers was tested in *C. sphinx* and *R. ferrumequinum* to ensure that the amplification efficiency was between 0.90 and 1.10. Similarly, a pair of bat-specific qRT-PCR primers for the reference gene *GAPDH* was designed.

### Polymerase Chain Reaction

Proximal promoter regions of *SLC2A2* in bats were amplified using two pairs of primers (see Table 4). The following polymerase chain reaction (PCR) protocol was used: pre-denaturation at 95 °C for 5 min, followed by 30 cycles of 30 sec at 94 °C, 30 sec at 45–50 °C and 20 sec at 72 °C, and a final elongation of 10 min at 72 °C. PCR fragments were then separated on 1% agarose gels and purified with a TaKaRa MiniBEST Agarose Gel DNA Extraction Kit (Takara, Japan). Products were then cloned into pGEM-T vectors (Promega, USA), propagated in DH5α or TOP10 competent cells (TIANGEN, China), and sequenced using Big Dye Terminator on an ABI 3730 DNA sequencer (Applied Biosystems, USA). *SLC2A2* proximal promoter sequences were amplified twice from one individual of each species for cloning.

### Multiple sequence aliment and analysis

The promoter sequence of human *SLC2A2* was retrieved from the Ensembl database and used as the reference for a multiple sequence alignment with the cloned partial *SLC2A2* proximal promoter sequences from bats using MUSCLE^35^. Bats were divided into three dietary groups, NWFBs, OWFBs, and non-frugivorous bats, based on diet. To assess the pairwise evolutionary divergence of these partial proximal promoter sequences among the three bat groups, the best-fitting nucleotide substitution model was identified using jModelTest^36^ under the Bayesian information criterion (BIC). This model was then used to establish the parameter setting for the Tajima’s relative rate and molecular clock tests with MEGA 5.0^37^. Transcription factor binding sites in the *SLC2A2* proximal promoter sequences from bats were predicted using an online transcription factor binding prediction tool (http://jaspar.genereg.net)^26^.

### Quantitative Real-Time reverse transcriptase PCR

Quantitative real-time reverse transcriptase PCR (qRT-PCR) was conducted on a 7300 Real-time PCR system (Applied Biosystems, USA) with two pairs of primers, one each to amplify *SLC2A2* and *GAPDH* (see in Table 4). All amplifications were repeated twice. Amplifications were carried out with SYBR^®^ Premix Ex Taq^TM^ Kit (Takara, Japan) at a final volume of 20 μl, containing 1 μl cDNA sample, 10 μl SYBR Premix ExTaq (Takara, Japan), 0.4 μl ROX Reference Dye, 1 μl of each primer, and 6.6 μl ddH_2_O. Reactions without cDNA template were used as controls. PCR amplifications were performed in triplicate wells under the following conditions: 30 sec at 95 °C, followed by 40 cycles of 5 sec at 95 °C and 31 sec at 60 °C. The dissociation curve analysis was performed after each assay to determine target specificity.

## Additional Information

**How to cite this article**: Meng, F. *et al.* Bats: Body mass index, forearm mass index, blood glucose levels and *SLC2A2* genes for diabetes. *Sci. Rep.* **6**, 29960; doi: 10.1038/srep29960 (2016).

## Supplementary Material

Supplementary Information

## Figures and Tables

**Table 1:** Taxonomy of bats used in this study, with accession numbers for the partial *SLC2A2* promoter sequences.

Taxonomy	Genbank common name	Species name	Accession Number
Suborder Yinpterochiroptera			
Family Pteropodidae			
Subfamily Cynopterinae	greater short-nosed fruit bat	*Cynopterus sphinx*	KT961113
Subfamily Rousettinae	Leschenault’s rousette	*Rousettus leschenaulti*	KT961116
	lesser dawn bat	*Eonycteris spelaea*	KT961114
Subfamily Nyctimeninae	unstriped tube-nosed bat	*Paranyctimene raptor*	KT961115
Subfamily Macroglossinae	southern blossom bat	*Syconycteris australis*	KT961117
Family Hipposideridae	great roundleaf bat	*Hipposideros armiger*	KT961103
	Pratt’s roundleaf bat	*H. pratti*	KT961104
Family Rhinolophidae	greater horseshoe bat	*Rhinolophus ferrumequinum*	KT961105
Suborder Yangochiroptera			
Family Phyllostomidae			
Subfamily Glossophaginae	Geoffroy’s tailless bat	*Anoura geoffroyi*	KT961112
Subfamily Carolliinae	Seba’s short-tailed bat	*Carollia perspicillata*	KT961110
	Sowell’s short-tailed bat	*C. sowelli*	KT961109
Subfamily Desmodontinae	common vampire bat	*Desmodus rotundus*	KT961108
Subfamily Stenodermatinae	great fruit-eating bat	*Artibeus lituratus*	KT961111
Family Rhinopomatidae	lesser mouse-tailed bat	*Rhinopoma hardwickii*	KT961106
Family Vespertilionidae	Rickett’s big-footed Myotis	*Myotis ricketti*	KU162944
Family Mormoopidae	ghost-faced bat	*Mormoops megalophylla*	KT961107

**Table 2:** Predicted transcription factor binding sites in bat *SLC2A2* promoters.

Species name	Diet	Predicted binding factor (and binding sites)
*M. ricketti*	Insectivorous	**MZF1(TGGGGA)** Nkx2-5(var.2) (AACCACTCAAG) SOX10 (CTTTGT)
*R. ferrumequinum*	Insectivorous	**Ahr::Arnt (TGCGTG)** MEIS1 (CTGACAG) **MZF1**, Nkx2-5(var.2), and SOX10
*H. armiger*	Insectivorous	MAFG::NFE2L1(CATGAC) MEIS1(ATGACAG) ZNF354C(CTCCAC) **MZF1**, Nkx2-5(var.2), and SOX10
*H. pratti*	Insectivorous	Comeplete the same as *H. armiger*
*R. hardwickii*	Insectivorous	**MAFG::NFE2L1(GATGAC) MEIS1(ATGACAG)** Nr2e3(CAAGCTT) MEIS1, **MZF1,** and Nkx2-5(var.2)
*M. megalophylla*	Insectivorous	None
*D. rotundus*	Sanguivorous	SOX10
*C. sowelli*	NWFBs	Nkx2-5(var.2) and SOX10
*C. perspicillata*	NWFBs	Nkx2-5(var.2) and SOX10
*A. lituratus*	NWFBs	**SNAI2(GGCAGGTGC) MZF1**, Nkx2-5(var.2) and SOX10
*A. geoffroyi*	NWFBs	Nkx2-5(var.2)
*E. spelaea*	OWFBs	FOXL1(ATAAACA) MAFG::NFE2L1(CATGAC), **MZF1**, and SOX10
*S. australis*	OWFBs	FOXL1, MAFG::NFE2L1, MEIS1, and SOX10
*C. sphinx*	OWFBs	FOXL1, MEIS1, and SOX10
*R. leschenaulti*	OWFBs	MAFG::NFE2L1, MEIS1,and SOX10
*P. raptor*	OWFBs	MAFG::NFE2L1, MEIS1, **MZF1**, SOX10

Promoter sequences from sixteen bats, including six insectivorous bats, one sanguivorous bat, four New World fruit-eating bats (NWFBs), and five Old World fruit-eating bats (OWFBs) were examined. Binding factors, and their predicted sites, in the cloned (+) or complementary (−) strands are shown in normal or bold font, respectively. Binding sites are only shown for their first appearance.

**Table 3:** Bases homologous to the human promoter SNPs rs5393 and rs5394 in *SLC2A2* promoter sequences of bats.

Species	Diet	(sub)Famliy	Bases homologous to SNP rs5393	Bases homologous to SNP rs5394
human	Omnivorous	Hominidae	A (Susceptible), C (Normal)	C (Susceptible), T (Normal)
*R. ferrumequinum*	Insectivorous	Rhinolophidae	C	C
*H. armiger*	Insectivorous	Hipposideridae	C	C
*H. pratti*	Insectivorous	Hipposideridae	C	C
*M. fuscus*	Insectivorous	Miniopteridae	C	C
*M. ricketti*	Insectivorous	Vespertilionidae	T	C
*R. hardwickii*	Insectivorous	Rhinopomatidae	C	C
*D. rotundus*	Sanguivorous	Desmodontinae	A	C
*C. sowelli*	Frugivorous	Carolliinae	A	C
*C. perspicillata*	Frugivorous	Carolliinae	A	C
*A. geoffroyi*	Frugivorous	Glossophaginae	A	C
*A.lituratus*	Frugivorous	Glossophaginae	A	G
*E. spelaea*	Frugivorous	Rousettinae	C	C
*S. australis*	Frugivorous	Macroglossinae	C	C
*C. sphinx*	Frugivorous	Cynopterinae	C	C
*R. leschenaulti*	Frugivorous	Rousettinae	C	C
*P. raptor*	Frugivorous	Nyctimeninae	C	C

**Table 4:** Sequences of primers used in this study.

**Primer**	**Sequence (5′ → 3′)**	**Tm (°C)**	**Product**	**Application**
Glut2-pmt-F1	GCTTAAGTTCACACCACCAG	57.8	~390 bp	Partial promoter sequences of *SLC2A2*
Glut2-pmt-R1	ATTGATGGAAATTTAATCAATA	47.0		
Glut2-pmt-F2	GAGATTCAAACCTAGGTTCA	53.7	~300 bp	Partial promoter sequences of *SLC2A2*
Glut2-pmt-R2	AGAGAAAATAGGAGCAGGTC	55.8		
Glut2-CDS-F	TCACCAACTCCAGCTACCGACA	59.4	257 bp	For amplifying partial coding region to design qRT-PCR primers of *SLC2A2*
Glut2-CDS-R	GTCGTCCTGCCTTCTCCACAAG	59.2		
Glut2-Q-F	CCGACAGCCTATTCTAGTGGCATTG	60.2	197 bp	For quantitative real-time PCR of *SLC2A2*
Glut2-Q-R	TGTTGATGGCACCAACTCCGATG	59.8		
GAPDH-Q-F	TCACCACCATGGAGAAGGC	59.7	252 bp	For quantitative real-time PCR of reference gene *GAPDH*
GAPDH-Q-R	GCTAAGCAGTTGGTGGTGCA	59.8		
