# Supplementary material for: Bats: Body mass index, forearm mass index, blood glucose levels and SLC2A2 genes for diabetes
Source: Sci Rep. 2016 Jul 21;6:29960. doi: 10.1038/srep29960 (PMC4954980; doi:10.1038/srep29960)
Supplement: Supplementary Information [file srep29960-s1.doc]

Bats: Body mass index, forearm mass index, blood glucose levels and *SLC2A2* genes for diabetes

Fanxing Meng^1^, Lei Zhu^1, 2, 3^, Wenjie Huang^1^, David M. Irwin^4^, Shuyi Zhang^5, 6, *^

^1^Institute of Molecular Ecology and Evolution, East China Normal University, Shanghai 200062, China

^2^State Key Laboratory of Genetic Resources and Evolution, Kunming Institute of Zoology, Chinese Academy of Sciences, Kunming 650223, China

^3^Kunming College of Life Science, University of Chinese Academy of Sciences, Kunming 650204, China

^4^Department of Laboratory Medicine and Pathobiology, University of Toronto, Toronto M5S 2E8, Canada

^5^Key Laboratory of Zoonosis of Liaoning Province, College of Animal Science and Veterinary Medicine, Shenyang Agricultural University, Shenyang 110866, China

^6^State Key Laboratory of Estuarine and Coastal Research, Institute of Estuarine and Coastal Research, East China Normal University, Shanghai 200062, China

*Correspondence should be addressed to S. Z. (email: [szhang_sau@126.com](mailto:szhang_sau@126.com)).

Appendix S1. Data retrieved on species used in this study. Data include species name, dietary, body weights, forearm lengths, forearm mass indexes (FMI), full body lengths (full length), body mass indexes (BMI), and diets. Missing data are shown as blanks. Data are alphabetically ordered by species names.

| Species name | Body weight | Forearm length | FMI | Full length | BMI | Diet |
| --- | --- | --- | --- | --- | --- | --- |
|  | g | mm | kg/m^2^ | mm | kg/m^2^ |  |
| *Acerodon celebensis* | 384.8 | 133.5 | 21.6 | 201.3 | 9.5 | Frugivore |
| *Acerodon humilis* | 352.4 | 140.0 | 18.0 |  |  | Frugivore |
| *Acerodon jubatus* | 1090.0 | 188.0 | 30.8 |  |  | Frugivore |
| *Acerodon leucotis* | 352.1 | 140.0 | 18.0 |  |  | Frugivore |
| *Acerodon mackloti* | 467.9 | 145.5 | 22.1 |  |  | Frugivore |
| *Aethalops aequalis* | 12.8 | 44.0 | 6.6 |  |  | Frugivore |
| *Aethalops alecto* | 15.0 | 46.5 | 6.9 |  |  | Frugivore |
| *Alionycteris paucidentata* | 16.2 | 44.6 | 8.2 | 63.8 | 4.0 | Frugivore |
| *Ametrida centurio* | 10.6 | 29.0 | 12.6 |  |  | Frugivore |
| *Amorphochilus schnablii* | 7.2 | 36.0 | 5.6 |  |  | Insectivore |
| *Anoura caudifera* | 10.8 | 36.3 | 8.2 | 58.3 | 3.2 | Insectivore |
| *Anoura cultrata* | 17.4 | 42.8 | 9.5 |  |  | Insectivore |
| *Anoura geoffroyi* | 15.2 | 42.7 | 8.3 |  |  | Insectivore |
| *Anoura latidens* | 15.1 | 43.0 | 8.1 |  |  | Insectivore |
| *Anthops ornatus* | 17.9 | 49.5 | 7.3 |  |  | Insectivore |
| *Antrozous dubiaquercus* | 22.2 | 53.3 | 7.8 |  |  | Insectivore |
| *Antrozous pallidus* | 22.2 | 54.5 | 7.5 |  |  | Insectivore |
| *Aproteles bulmerae* | 623.9 | 166.2 | 22.6 | 245.5 | 10.4 | Frugivore |
| *Ardops nichollsi* | 19.2 | 48.6 | 8.1 |  |  | Frugivore |
| *Ariteus flavescens* | 10.1 | 40.5 | 6.2 |  |  | Frugivore |
| *Artibeus amplus* | 61.0 | 70.5 | 12.3 |  |  | Frugivore |
| *Artibeus anderseni* | 6.9 | 35.5 | 5.5 |  |  | Frugivore |
| *Artibeus aztecus* | 20.8 | 44.4 | 10.6 |  |  | Frugivore |
| *Artibeus cinereus* | 12.7 | 39.0 | 8.3 |  |  | Frugivore |
| *Artibeus concolor* | 19.7 | 47.3 | 8.8 |  |  | Frugivore |
| *Artibeus fraterculus* | 25.5 | 56.0 | 8.1 |  |  | Frugivore |
| *Artibeus hartii* | 17.0 | 39.8 | 10.7 |  |  | Frugivore |
| *Artibeus hirsutus* | 40.4 | 55.9 | 12.9 |  |  | Frugivore |
| *Artibeus jamaicensis* | 43.6 | 60.3 | 12.0 | 78.3 | 7.1 | Frugivore |
| *Artibeus lituratus* | 59.3 | 70.3 | 12.0 | 95.4 | 6.5 | Frugivore |
| *Artibeus toltecus* | 15.5 | 39.5 | 9.9 |  |  | Frugivore |
| *Asellia patrizii* | 8.7 | 38.5 | 5.9 |  |  | Insectivore |
| *Asellia tridens* | 12.9 | 51.3 | 4.9 | 56.0 | 4.1 | Insectivore |
| *Aselliscus stoliczkanus* | 6.1 | 41.5 | 3.5 |  |  | Insectivore |
| *Aselliscus tricuspidatus* | 4.1 | 40.5 | 2.5 |  |  | Insectivore |
| *Balantiopteryx infusca* | 9.1 | 39.0 | 6.0 |  |  | Insectivore |
| *Balantiopteryx io* | 4.0 | 37.0 | 2.9 | 40.2 | 2.5 | Insectivore |
| *Balantiopteryx plicata* | 6.6 | 42.2 | 3.7 |  |  | Insectivore |
| *Balionycteris maculata* | 14.4 | 41.0 | 8.6 |  |  | Frugivore |
| *Barbastella barbastellus* | 8.3 | 38.5 | 5.6 |  |  | Insectivore |
| *Barbastella leucomelas* | 15.1 | 41.5 | 8.7 |  |  | Insectivore |
| *Brachyphylla cavernarum* | 45.5 | 66.2 | 10.4 | 81.3 | 6.9 | Omnivore |
| *Brachyphylla nana* | 37.3 | 58.3 | 11.0 | 84.2 | 5.3 | Frugivore |
| *Cardioderma cor* | 26.5 | 54.0 | 9.1 |  |  | Insectivore |
| *Carollia brevicauda* | 14.9 | 40.3 | 9.1 |  |  | Frugivore |
| *Carollia castanea* | 13.1 | 36.2 | 10.0 |  |  | Frugivore |
| *Carollia perspicillata* | 19.2 | 42.5 | 10.6 | 60.0 | 5.3 | Omnivore |
| *Carollia subrufa* | 15.8 | 39.4 | 10.2 |  |  | Frugivore |
| *Casinycteris argynnis* | 28.3 | 56.0 | 9.0 |  |  | Frugivore |
| *Centronycteris maximiliani* | 23.0 | 45.5 | 11.1 |  |  | Insectivore |
| *Centurio senex* | 23.1 | 44.0 | 11.9 |  |  | Frugivore |
| *Chaerephon aloysiisabaudiae* | 19.5 | 51.0 | 7.5 |  |  | Insectivore |
| *Chaerephon ansorgei* | 14.5 | 46.0 | 6.9 |  |  | Insectivore |
| *Chaerephon bemmeleni* | 12.4 | 43.5 | 6.5 |  |  | Insectivore |
| *Chaerephon bivittata* | 15.4 | 49.0 | 6.4 |  |  | Insectivore |
| *Chaerephon chapini* | 7.5 | 36.5 | 5.6 |  |  | Insectivore |
| *Chaerephon gallagheri* | 8.1 | 37.5 | 5.8 |  |  | Insectivore |
| *Chaerephon jobensis* | 20.7 | 46.5 | 9.6 |  |  | Insectivore |
| *Chaerephon johorensis* | 15.7 | 47.2 | 7.0 |  |  | Insectivore |
| *Chaerephon major* | 15.2 | 44.9 | 7.5 |  |  | Insectivore |
| *Chaerephon nigeriae* | 20.1 | 47.0 | 9.1 |  |  | Insectivore |
| *Chaerephon plicata* | 21.8 | 46.4 | 10.1 |  |  | Insectivore |
| *Chaerephon pumila* | 11.0 | 39.0 | 7.2 |  |  | Insectivore |
| *Chaerephon russata* | 17.0 | 44.5 | 8.6 |  |  | Insectivore |
| *Chaerephon tomensis* | 7.5 | 38.6 | 5.0 |  |  | Insectivore |
| *Chalinolobus dwyeri* | 8.7 | 40.0 | 5.5 |  |  | Insectivore |
| *Chalinolobus gouldii* | 14.2 | 43.9 | 7.4 |  |  | Insectivore |
| *Chalinolobus morio* | 8.9 | 37.0 | 6.5 |  |  | Insectivore |
| *Chalinolobus nigrogriseus* | 8.8 | 35.0 | 7.2 |  |  | Insectivore |
| *Chalinolobus picatus* | 5.9 | 33.5 | 5.2 |  |  | Insectivore |
| *Chalinolobus tuberculatus* | 10.5 | 41.0 | 6.2 |  |  | Insectivore |
| *Chalinolobus variegatus* | 11.3 | 43.5 | 5.9 |  |  | Insectivore |
| *Cheiromeles torquatus* | 169.4 | 81.5 | 25.5 |  |  | Insectivore |
| *Chiroderma doriae* | 19.9 | 52.5 | 7.2 |  |  | Frugivore |
| *Chiroderma improvisum* | 35.4 | 57.7 | 10.6 |  |  | Frugivore |
| *Chiroderma salvini* | 26.3 | 47.5 | 11.7 |  |  | Frugivore |
| *Chiroderma trinitatum* | 13.9 | 36.4 | 10.5 |  |  | Frugivore |
| *Chiroderma villosum* | 23.8 | 45.5 | 11.5 | 64.0 | 5.8 | Frugivore |
| *Chironax melanocephalus* | 17.7 | 45.5 | 8.5 |  |  | Frugivore |
| *Choeroniscus godmani* | 7.9 | 34.0 | 6.8 |  |  | Insectivore |
| *Choeroniscus minor* | 8.6 | 35.5 | 6.8 |  |  | Insectivore |
| *Choeroniscus periosus* | 10.5 | 41.0 | 6.2 |  |  | Insectivore |
| *Choeronycteris mexicana* | 17.3 | 44.5 | 8.7 | 66.0 | 4.0 | Insectivore |
| *Chrotopterus auritus* | 78.3 | 82.6 | 11.5 | 103.0 | 7.4 | Omnivore |
| *Cistugo lesueuri* | 6.4 | 34.5 | 5.4 |  |  | Insectivore |
| *Cistugo seabrae* | 5.4 | 32.5 | 5.1 |  |  | Insectivore |
| *Cloeotis percivali* | 4.2 | 34.5 | 3.5 |  |  | Insectivore |
| *Coelops frithi* | 7.5 | 41.0 | 4.5 |  |  | Insectivore |
| *Coelops robinsoni* | 6.5 | 35.0 | 5.3 |  |  | Insectivore |
| *Coleura afra* | 10.7 | 48.0 | 4.6 |  |  | Insectivore |
| *Coleura seychellensis* | 10.6 | 54.3 | 3.6 | 60.0 | 3.0 | Insectivore |
| *Cormura brevirostris* | 9.3 | 46.5 | 4.3 |  |  | Insectivore |
| *Craseonycteris thonglongyai* | 2.0 | 24.1 | 3.4 | 31.0 | 2.1 | Insectivore |
| *Cynopterus brachyotis* | 33.9 | 64.2 | 8.2 |  |  | Frugivore |
| *Cynopterus horsfieldii* | 56.0 | 73.5 | 10.4 |  |  | Frugivore |
| *Cynopterus sphinx* | 44.7 | 71.0 | 8.9 | 99.0 | 4.6 | Frugivore |
| *Cynopterus titthaecheilus* | 58.3 | 76.9 | 9.9 |  |  | Frugivore |
| *Cyttarops alecto* | 5.3 | 47.0 | 2.4 |  |  | Insectivore |
| *Desmalopex leucopterus* | 343.8 | 139.5 | 17.7 |  |  | Frugivore |
| *Desmodus rotundus* | 33.2 | 58.3 | 9.8 | 78.0 | 5.5 | Sanguivore |
| *Diaemus youngi* | 36.7 | 50.8 | 14.2 |  |  | Sanguivore |
| *Diclidurus albus* | 16.6 | 66.0 | 3.8 | 70.0 | 3.4 | Insectivore |
| *Diclidurus ingens* | 12.3 | 71.5 | 2.4 |  |  | Insectivore |
| *Diclidurus isabellus* | 12.6 | 54.0 | 4.3 |  |  | Insectivore |
| *Diclidurus scutatus* | 13.6 | 54.5 | 4.6 |  |  | Insectivore |
| *Diphylla ecaudata* | 28.1 | 55.0 | 9.3 | 79.0 | 4.5 | Sanguivore |
| *Dobsonia anderseni* | 234.0 | 122.6 | 15.6 |  |  | Frugivore |
| *Dobsonia beauforti* | 165.4 | 105.7 | 14.8 | 161.3 | 6.4 | Frugivore |
| *Dobsonia chapmani* | 269.6 | 127.5 | 16.6 |  |  | Frugivore |
| *Dobsonia crenulata* | 218.2 | 122.4 | 14.6 | 178.1 | 6.9 | Frugivore |
| *Dobsonia emersa* | 201.1 | 115.6 | 15.0 | 164.3 | 7.4 | Frugivore |
| *Dobsonia exoleta* | 302.0 | 114.8 | 22.9 | 202.7 | 7.4 | Frugivore |
| *Dobsonia inermis* | 152.6 | 107.3 | 13.3 | 174.0 | 5.0 | Frugivore |
| *Dobsonia minor* | 86.0 | 80.0 | 13.4 |  |  | Frugivore |
| *Dobsonia moluccensis* | 447.6 | 138.7 | 23.3 |  |  | Frugivore |
| *Dobsonia pannietensis* | 239.2 | 110.8 | 19.5 | 186.2 | 6.9 | Frugivore |
| *Dobsonia peroni* | 226.5 | 115.1 | 17.1 |  |  | Frugivore |
| *Dobsonia praedatrix* | 178.7 | 116.5 | 13.2 | 161.1 | 6.9 | Frugivore |
| *Dobsonia viridis* | 240.7 | 115.0 | 18.2 |  |  | Frugivore |
| *Dyacopterus brooksi* | 74.7 | 81.5 | 11.2 |  |  | Frugivore |
| *Dyacopterus spadiceus* | 81.1 | 79.7 | 12.8 | 129.3 | 4.9 | Frugivore |
| *Ectophylla alba* | 5.6 | 28.7 | 6.7 |  |  | Frugivore |
| *Eidolon dupreanum* | 297.6 | 127.7 | 18.2 | 202.5 | 7.3 | Frugivore |
| *Eidolon helvum* | 254.6 | 120.5 | 17.5 | 182.6 | 7.6 | Frugivore |
| *Emballonura alecto* | 5.3 | 46.0 | 2.5 |  |  | Insectivore |
| *Emballonura atrata* | 4.6 | 38.2 | 3.1 | 57.5 | 1.4 | Insectivore |
| *Emballonura beccarii* | 4.3 | 41.0 | 2.6 |  |  | Insectivore |
| *Emballonura dianae* | 13.2 | 44.5 | 6.7 |  |  | Insectivore |
| *Emballonura furax* | 15.9 | 47.5 | 7.1 |  |  | Insectivore |
| *Emballonura monticola* | 5.4 | 44.0 | 2.8 |  |  | Insectivore |
| *Emballonura raffrayana* | 5.6 | 42.5 | 3.1 |  |  | Insectivore |
| *Emballonura semicaudata* | 6.3 | 46.5 | 2.9 | 47.0 | 2.8 | Insectivore |
| *Eonycteris major* | 70.7 | 79.8 | 11.1 |  |  | Frugivore |
| *Eonycteris spelaea* | 58.7 | 69.0 | 12.3 | 123.8 | 3.8 | Frugivore |
| *Epomophorus angolensis* | 88.8 | 86.5 | 11.9 |  |  | Frugivore |
| *Epomophorus crypturus* | 95.0 | 80.5 | 14.7 |  |  | Frugivore |
| *Epomophorus gambianus* | 134.6 | 85.3 | 18.5 | 159.4 | 5.3 | Frugivore |
| *Epomophorus grandis* | 49.4 | 63.6 | 12.2 | 136.0 | 2.7 | Frugivore |
| *Epomophorus labiatus* | 69.7 | 65.2 | 16.4 | 94.3 | 7.8 | Frugivore |
| *Epomophorus wahlbergi* | 93.6 | 80.7 | 14.4 | 131.7 | 5.4 | Frugivore |
| *Epomops buettikoferi* | 135.7 | 90.2 | 16.7 |  |  | Frugivore |
| *Epomops dobsoni* | 122.1 | 84.5 | 17.1 |  |  | Frugivore |
| *Epomops franqueti* | 119.0 | 89.4 | 14.9 | 135.0 | 6.5 | Frugivore |
| *Eptesicus baverstocki* | 4.3 | 30.1 | 4.7 |  |  | Insectivore |
| *Eptesicus bobrinskoi* | 7.5 | 36.0 | 5.8 |  |  | Insectivore |
| *Eptesicus bottae* | 15.7 | 43.0 | 8.5 |  |  | Insectivore |
| *Eptesicus brasiliensis* | 9.2 | 43.0 | 5.0 |  |  | Insectivore |
| *Eptesicus brunneus* | 6.9 | 35.5 | 5.5 |  |  | Insectivore |
| *Eptesicus capensis* | 6.0 | 31.5 | 6.0 |  |  | Insectivore |
| *Eptesicus diminutus* | 6.0 | 34.5 | 5.0 |  |  | Insectivore |
| *Eptesicus dimissus* | 13.0 | 42.0 | 7.4 |  |  | Insectivore |
| *Eptesicus douglasorum* | 5.0 | 36.0 | 3.9 |  |  | Insectivore |
| *Eptesicus floweri* | 7.2 | 36.0 | 5.6 |  |  | Insectivore |
| *Eptesicus furinalis* | 7.7 | 39.2 | 5.0 |  |  | Insectivore |
| *Eptesicus fuscus* | 17.5 | 46.7 | 8.0 |  |  | Insectivore |
| *Eptesicus guadeloupensis* | 19.0 | 50.5 | 7.4 |  |  | Insectivore |
| *Eptesicus hottentotus* | 30.3 | 49.5 | 12.4 |  |  | Insectivore |
| *Eptesicus innoxius* | 7.5 | 36.5 | 5.6 |  |  | Insectivore |
| *Eptesicus kobayashii* | 14.5 | 46.0 | 6.9 |  |  | Insectivore |
| *Eptesicus melckorum* | 7.2 | 36.0 | 5.6 |  |  | Insectivore |
| *Eptesicus nasutus* | 7.9 | 37.0 | 5.8 |  |  | Insectivore |
| *Eptesicus nilssoni* | 10.7 | 40.2 | 6.6 |  |  | Insectivore |
| *Eptesicus pachyotis* | 8.1 | 38.5 | 5.5 |  |  | Insectivore |
| *Eptesicus pumilus* | 5.4 | 31.5 | 5.4 |  |  | Insectivore |
| *Eptesicus regulus* | 5.1 | 30.1 | 5.6 |  |  | Insectivore |
| *Eptesicus rendalli* | 6.4 | 34.5 | 5.4 |  |  | Insectivore |
| *Eptesicus serotinus* | 23.1 | 50.3 | 9.1 | 68.0 | 5.0 | Insectivore |
| *Eptesicus somalicus* | 3.5 | 28.9 | 4.2 |  |  | Insectivore |
| *Eptesicus tatei* | 13.7 | 45.0 | 6.7 |  |  | Insectivore |
| *Eptesicus tenuipinnis* | 5.4 | 31.0 | 5.6 | 37.0 | 3.9 | Insectivore |
| *Eptesicus vulturnus* | 3.8 | 26.9 | 5.2 |  |  | Insectivore |
| *Erophylla bombifrons* | 16.3 | 47.1 | 7.3 |  |  | Omnivore |
| *Erophylla sezekorni* | 15.9 | 47.5 | 7.0 |  |  | Omnivore |
| *Euderma maculatum* | 16.2 | 49.5 | 6.6 |  |  | Insectivore |
| *Eudiscopus denticulus* | 7.2 | 36.0 | 5.6 |  |  | Insectivore |
| *Eumops auripendulus* | 28.5 | 61.5 | 7.5 |  |  | Insectivore |
| *Eumops bonariensis* | 12.2 | 40.1 | 7.6 |  |  | Insectivore |
| *Eumops dabbenei* | 67.3 | 76.5 | 11.5 |  |  | Insectivore |
| *Eumops glaucinus* | 36.2 | 59.7 | 10.2 |  |  | Insectivore |
| *Eumops hansae* | 15.5 | 38.3 | 10.5 |  |  | Insectivore |
| *Eumops maurus* | 20.7 | 52.0 | 7.6 |  |  | Insectivore |
| *Eumops perotis* | 51.0 | 75.5 | 8.9 |  |  | Insectivore |
| *Eumops underwoodi* | 58.7 | 71.2 | 11.6 |  |  | Insectivore |
| *Furipterus horrens* | 3.2 | 35.5 | 2.5 |  |  | Insectivore |
| *Glauconycteris alboguttata* | 9.1 | 39.0 | 6.0 |  |  | Insectivore |
| *Glauconycteris argentata* | 9.3 | 40.5 | 5.7 |  |  | Insectivore |
| *Glauconycteris beatrix* | 7.5 | 37.0 | 5.5 |  |  | Insectivore |
| *Glauconycteris egeria* | 8.4 | 38.0 | 5.8 |  |  | Insectivore |
| *Glauconycteris gleni* | 10.8 | 40.0 | 6.8 |  |  | Insectivore |
| *Glauconycteris kenyacola* | 7.2 | 40.5 | 4.4 |  |  | Insectivore |
| *Glauconycteris poensis* | 6.9 | 37.5 | 4.9 |  |  | Insectivore |
| *Glauconycteris superba* | 15.0 | 46.5 | 6.9 |  |  | Insectivore |
| *Glischropus javanus* | 5.4 | 32.5 | 5.1 |  |  | Insectivore |
| *Glischropus tylopus* | 4.6 | 29.0 | 5.5 |  |  | Insectivore |
| *Glossophaga commissarisi* | 9.2 | 34.4 | 7.7 |  |  | Frugivore |
| *Glossophaga leachii* | 10.2 | 35.7 | 8.0 |  |  | Frugivore |
| *Glossophaga longirostris* | 13.3 | 38.5 | 9.0 |  |  | Frugivore |
| *Glossophaga morenoi* | 8.5 | 34.5 | 7.2 |  |  | Frugivore |
| *Glossophaga soricina* | 10.0 | 35.7 | 7.8 | 49.0 | 4.2 | Frugivore |
| *Haplonycteris fischeri* | 18.3 | 50.6 | 7.1 |  |  | Frugivore |
| *Harpiocephalus harpia* | 13.7 | 44.9 | 6.8 |  |  | Insectivore |
| *Harpiocephalus mordax* | 20.1 | 51.5 | 7.6 |  |  | Insectivore |
| *Harpyionycteris celebensis* | 116.8 | 90.3 | 14.3 | 144.2 | 5.6 | Frugivore |
| *Harpyionycteris whiteheadi* | 135.5 | 85.5 | 18.5 | 152.2 | 5.8 | Frugivore |
| *Hesperoptenus blanfordi* | 6.9 | 26.5 | 9.8 |  |  | Insectivore |
| *Hesperoptenus doriae* | 9.7 | 40.0 | 6.1 |  |  | Insectivore |
| *Hesperoptenus gaskelli* | 9.4 | 39.5 | 6.0 |  |  | Insectivore |
| *Hesperoptenus tickelli* | 16.3 | 55.0 | 5.4 |  |  | Insectivore |
| *Hesperoptenus tomesi* | 20.7 | 52.0 | 7.6 |  |  | Insectivore |
| *Hipposideros abae* | 31.9 | 60.5 | 8.7 |  |  | Insectivore |
| *Hipposideros armiger* | 50.0 | 89.5 | 6.2 |  |  | Insectivore |
| *Hipposideros ater* | 5.9 | 39.0 | 3.9 |  |  | Insectivore |
| *Hipposideros beatus* | 6.7 | 43.5 | 3.5 |  |  | Insectivore |
| *Hipposideros bicolor* | 8.4 | 43.8 | 4.4 |  |  | Insectivore |
| *Hipposideros breviceps* | 13.2 | 44.5 | 6.7 |  |  | Insectivore |
| *Hipposideros caffer* | 9.5 | 46.5 | 4.4 | 55.0 | 3.1 | Insectivore |
| *Hipposideros calcaratus* | 19.0 | 50.5 | 7.4 |  |  | Insectivore |
| *Hipposideros camerunensis* | 59.0 | 75.0 | 10.5 |  |  | Insectivore |
| *Hipposideros cervinus* | 8.5 | 46.0 | 4.0 |  |  | Insectivore |
| *Hipposideros cineraceus* | 3.8 | 34.9 | 3.2 |  |  | Insectivore |
| *Hipposideros commersoni* | 90.0 | 96.0 | 9.8 | 127.5 | 5.5 | Insectivore |
| *Hipposideros coronatus* | 15.0 | 46.5 | 6.9 |  |  | Insectivore |
| *Hipposideros corynophyllus* | 15.1 | 48.5 | 6.4 |  |  | Insectivore |
| *Hipposideros coxi* | 21.8 | 53.0 | 7.8 |  |  | Insectivore |
| *Hipposideros crumeniferus* | 21.8 | 53.0 | 7.8 |  |  | Insectivore |
| *Hipposideros curtus* | 13.2 | 44.5 | 6.7 |  |  | Insectivore |
| *Hipposideros cyclops* | 32.9 | 64.7 | 7.9 | 76.0 | 5.7 | Insectivore |
| *Hipposideros diadema* | 46.9 | 88.7 | 6.0 |  |  | Insectivore |
| *Hipposideros dinops* | 116.1 | 95.0 | 12.9 |  |  | Insectivore |
| *Hipposideros doriae* | 4.2 | 37.5 | 3.0 |  |  | Insectivore |
| *Hipposideros dyacorum* | 10.1 | 40.5 | 6.2 |  |  | Insectivore |
| *Hipposideros fuliginosus* | 31.1 | 60.0 | 8.6 |  |  | Insectivore |
| *Hipposideros fulvus* | 8.8 | 40.9 | 5.3 |  |  | Insectivore |
| *Hipposideros galeritus* | 10.2 | 46.0 | 4.8 |  |  | Insectivore |
| *Hipposideros halophyllus* | 4.0 | 37.0 | 2.9 |  |  | Insectivore |
| *Hipposideros inexpectatus* | 136.3 | 100.5 | 13.5 |  |  | Insectivore |
| *Hipposideros inornatus* | 25.9 | 71.1 | 5.1 |  |  | Insectivore |
| *Hipposideros jonesi* | 5.5 | 49.5 | 2.2 |  |  | Insectivore |
| *Hipposideros lamottei* | 24.9 | 55.5 | 8.1 |  |  | Insectivore |
| *Hipposideros lankadiva* | 44.8 | 83.5 | 6.4 |  |  | Insectivore |
| *Hipposideros larvatus* | 20.0 | 60.0 | 5.5 |  |  | Insectivore |
| *Hipposideros lekaguli* | 31.1 | 75.5 | 5.5 |  |  | Insectivore |
| *Hipposideros lylei* | 40.0 | 78.5 | 6.5 |  |  | Insectivore |
| *Hipposideros macrobullatus* | 10.8 | 41.5 | 6.3 |  |  | Insectivore |
| *Hipposideros maggietaylarae* | 16.1 | 59.0 | 4.6 |  |  | Insectivore |
| *Hipposideros marisae* | 10.1 | 40.5 | 6.2 |  |  | Insectivore |
| *Hipposideros megalotis* | 7.2 | 36.0 | 5.6 |  |  | Insectivore |
| *Hipposideros muscinus* | 14.1 | 45.5 | 6.8 |  |  | Insectivore |
| *Hipposideros obscurus* | 9.6 | 42.5 | 5.3 |  |  | Insectivore |
| *Hipposideros papua* | 19.0 | 50.5 | 7.4 |  |  | Insectivore |
| *Hipposideros pomona* | 6.2 | 40.5 | 3.8 |  |  | Insectivore |
| *Hipposideros pratti* | 84.4 | 85.0 | 11.7 |  |  | Insectivore |
| *Hipposideros pygmaeus* | 3.5 | 38.0 | 2.4 |  |  | Insectivore |
| *Hipposideros ridleyi* | 9.6 | 46.9 | 4.4 |  |  | Insectivore |
| *Hipposideros ruber* | 10.6 | 49.6 | 4.3 |  |  | Insectivore |
| *Hipposideros semoni* | 14.0 | 45.5 | 6.8 |  |  | Insectivore |
| *Hipposideros speoris* | 10.4 | 51.5 | 3.9 |  |  | Insectivore |
| *Hipposideros stenotis* | 12.0 | 43.5 | 6.3 |  |  | Insectivore |
| *Hipposideros turpis* | 33.3 | 73.5 | 6.2 |  |  | Insectivore |
| *Hipposideros wallastoni* | 6.9 | 42.5 | 3.8 |  |  | Insectivore |
| *Histiotus macrotus* | 11.0 | 48.5 | 4.7 |  |  | Insectivore |
| *Histiotus montanus* | 15.9 | 47.5 | 7.1 |  |  | Insectivore |
| *Histiotus velatus* | 11.3 | 47.0 | 5.1 |  |  | Insectivore |
| *Hylonycteris underwoodi* | 7.5 | 34.0 | 6.5 | 59.8 | 2.1 | Omnivore |
| *Hypsignathus monstrosus* | 337.0 | 127.5 | 20.7 | 226.5 | 6.6 | Frugivore |
| *Ia io* | 49.3 | 75.5 | 8.6 |  |  | Carnivore |
| *Idionycteris phyllotis* | 12.1 | 44.5 | 6.1 | 59.0 | 3.5 | Insectivore |
| *Kerivoula aerosa* | 7.8 | 37.0 | 5.7 |  |  | Insectivore |
| *Kerivoula africana* | 3.5 | 28.0 | 4.5 |  |  | Insectivore |
| *Kerivoula agnella* | 7.2 | 36.0 | 5.6 |  |  | Insectivore |
| *Kerivoula argentata* | 10.1 | 36.5 | 7.6 |  |  | Insectivore |
| *Kerivoula atrox* | 4.8 | 33.0 | 4.4 |  |  | Insectivore |
| *Kerivoula cuprosa* | 5.6 | 33.0 | 5.2 |  |  | Insectivore |
| *Kerivoula eriophora* | 3.5 | 28.0 | 4.5 |  |  | Insectivore |
| *Kerivoula flora* | 6.0 | 38.2 | 4.1 |  |  | Insectivore |
| *Kerivoula hardwickei* | 4.6 | 35.0 | 3.7 |  |  | Insectivore |
| *Kerivoula intermedia* | 3.7 | 28.5 | 4.5 |  |  | Insectivore |
| *Kerivoula jagorii* | 4.7 | 37.8 | 3.3 |  |  | Insectivore |
| *Kerivoula lanosa* | 6.7 | 30.0 | 7.4 |  |  | Insectivore |
| *Kerivoula minuta* | 2.0 | 27.0 | 2.8 |  |  | Insectivore |
| *Kerivoula muscina* | 5.3 | 32.5 | 5.0 |  |  | Insectivore |
| *Kerivoula myrella* | 7.2 | 36.0 | 5.6 |  |  | Insectivore |
| *Kerivoula papillosa* | 10.2 | 41.0 | 6.1 |  |  | Insectivore |
| *Kerivoula papuensis* | 6.3 | 37.6 | 4.5 |  |  | Insectivore |
| *Kerivoula pellucida* | 4.1 | 31.5 | 4.2 |  |  | Insectivore |
| *Kerivoula phalaena* | 3.0 | 28.0 | 3.9 |  |  | Insectivore |
| *Kerivoula picta* | 4.5 | 35.1 | 3.7 |  |  | Insectivore |
| *Kerivoula smithii* | 5.9 | 33.5 | 5.2 |  |  | Insectivore |
| *Kerivoula whiteheadi* | 3.3 | 30.0 | 3.7 |  |  | Insectivore |
| *Laephotis angolensis* | 6.1 | 34.0 | 5.3 |  |  | Insectivore |
| *Laephotis botswanae* | 7.3 | 36.0 | 5.6 |  |  | Insectivore |
| *Laephotis namibensis* | 8.7 | 38.5 | 5.9 |  |  | Insectivore |
| *Laephotis wintoni* | 6.1 | 38.5 | 4.1 |  |  | Insectivore |
| *Lampronycteris brachyotis* | 10.4 | 39.6 | 6.6 |  |  | Omnivore |
| *Lasionycteris noctivagans* | 11.0 | 41.3 | 6.5 |  |  | Insectivore |
| *Lasiurus borealis* | 12.3 | 40.7 | 7.4 |  |  | Insectivore |
| *Lasiurus castaneus* | 12.5 | 44.5 | 6.3 |  |  | Insectivore |
| *Lasiurus cinereus* | 27.1 | 54.4 | 9.1 | 75.3 | 4.8 | Insectivore |
| *Lasiurus ebenus* | 14.0 | 46.0 | 6.6 |  |  | Insectivore |
| *Lasiurus ega* | 12.2 | 47.5 | 5.4 |  |  | Insectivore |
| *Lasiurus egregius* | 17.4 | 49.0 | 7.3 |  |  | Insectivore |
| *Lasiurus intermedius* | 23.0 | 55.1 | 7.6 |  |  | Insectivore |
| *Latidens salimalii* | 50.0 | 67.5 | 11.0 | 106.4 | 4.4 | Frugivore |
| *Lavia frons* | 23.8 | 56.0 | 7.6 | 67.0 | 5.3 | Insectivore |
| *Leptonycteris curasoae* | 25.3 | 52.8 | 9.1 |  |  | Frugivore |
| *Leptonycteris nivalis* | 24.3 | 56.8 | 7.5 |  |  | Frugivore |
| *Leptonycteris yerbabuenae* | 22.2 | 53.4 | 7.8 |  |  | Frugivore |
| *Lichonycteris obscura* | 6.5 | 31.6 | 6.5 |  |  | Frugivore |
| *Lionycteris spurrelli* | 8.9 | 35.0 | 7.2 |  |  | Frugivore |
| *Lonchophylla bokermanni* | 9.7 | 40.0 | 6.1 |  |  | Frugivore |
| *Lonchophylla dekeyseri* | 7.2 | 36.0 | 5.6 |  |  | Frugivore |
| *Lonchophylla handleyi* | 14.5 | 46.0 | 6.9 |  |  | Frugivore |
| *Lonchophylla hesperia* | 8.7 | 38.5 | 5.9 |  |  | Frugivore |
| *Lonchophylla mordax* | 21.6 | 33.9 | 18.8 |  |  | Frugivore |
| *Lonchophylla robusta* | 13.7 | 42.5 | 7.6 |  |  | Frugivore |
| *Lonchophylla thomasi* | 7.1 | 32.5 | 6.7 |  |  | Frugivore |
| *Lonchorhina aurita* | 15.4 | 51.0 | 5.9 |  |  | Insectivore |
| *Lonchorhina fernandezi* | 11.6 | 42.5 | 6.4 |  |  | Insectivore |
| *Lonchorhina marinkelli* | 17.7 | 59.5 | 5.0 |  |  | Insectivore |
| *Lonchorhina orinocensis* | 9.0 | 43.0 | 4.9 |  |  | Insectivore |
| *Macroderma gigas* | 124.4 | 107.0 | 10.9 | 115.8 | 9.3 | Carnivore |
| *Macroglossus minimus* | 16.3 | 40.2 | 10.1 | 68.1 | 3.5 | Frugivore |
| *Macroglossus sobrinus* | 21.8 | 47.0 | 9.9 |  |  | Frugivore |
| *Macrophyllum macrophyllum* | 8.0 | 35.6 | 6.3 |  |  | Insectivore |
| *Macrotus californicus* | 11.8 | 50.6 | 4.6 |  |  | Insectivore |
| *Macrotus waterhousii* | 16.3 | 51.6 | 6.1 | 58.4 | 4.8 | Insectivore |
| *Megaderma lyra* | 39.3 | 69.5 | 8.1 | 74.0 | 7.2 | Carnivore |
| *Megaderma spasma* | 24.7 | 56.5 | 7.7 |  |  | Carnivore |
| *Megaerops ecaudatus* | 26.3 | 54.5 | 8.9 |  |  | Frugivore |
| *Megaerops kusnotoi* | 19.9 | 51.3 | 7.6 |  |  | Frugivore |
| *Megaerops niphanae* | 32.6 | 57.2 | 10.0 |  |  | Frugivore |
| *Megaerops wetmorei* | 18.7 | 49.5 | 7.6 |  |  | Frugivore |
| *Megaloglossus woermanni* | 16.7 | 41.8 | 9.6 | 40.6 | 10.1 | Frugivore |
| *Melonycteris melanops* | 47.6 | 60.0 | 13.2 | 94.1 | 5.4 | Frugivore |
| *Melonycteris woodfordi* | 36.2 | 53.7 | 12.6 | 88.3 | 4.6 | Frugivore |
| *Mesophylla macconnelli* | 6.9 | 31.6 | 6.9 |  |  | Frugivore |
| *Micronycteris daviesi* | 18.6 | 55.5 | 6.0 |  |  | Insectivore |
| *Micronycteris hirsuta* | 12.9 | 43.3 | 6.9 |  |  | Insectivore |
| *Micronycteris megalotis* | 6.4 | 34.0 | 5.5 |  |  | Insectivore |
| *Micronycteris minuta* | 6.9 | 34.0 | 6.0 |  |  | Insectivore |
| *Micronycteris nicefori* | 8.3 | 38.0 | 5.7 |  |  | Insectivore |
| *Micronycteris pusilla* | 6.1 | 34.0 | 5.3 |  |  | Insectivore |
| *Micronycteris schmidtorum* | 7.7 | 35.5 | 6.1 |  |  | Insectivore |
| *Micronycteris sylvestris* | 8.9 | 40.5 | 5.4 |  |  | Insectivore |
| *Mimetillus moloneyi* | 8.9 | 28.0 | 11.3 |  |  | Insectivore |
| *Miniopterus australis* | 7.4 | 40.5 | 4.5 |  |  | Insectivore |
| *Miniopterus fraterculus* | 7.4 | 42.5 | 4.1 |  |  | Insectivore |
| *Miniopterus fuscus* | 10.8 | 41.5 | 6.3 |  |  | Insectivore |
| *Miniopterus inflatus* | 14.9 | 48.1 | 6.4 |  |  | Insectivore |
| *Miniopterus magnater* | 14.1 | 50.0 | 5.7 |  |  | Insectivore |
| *Miniopterus robustior* | 10.5 | 41.0 | 6.2 |  |  | Insectivore |
| *Miniopterus schreibersi* | 11.5 | 46.0 | 5.4 |  |  | Insectivore |
| *Miniopterus tristis* | 15.2 | 51.0 | 5.8 |  |  | Insectivore |
| *Mirimiri acrodonta* | 257.5 | 118.0 | 18.5 | 186.8 | 7.4 | Frugivore |
| *Molossops abrasus* | 35.4 | 43.5 | 18.7 |  |  | Insectivore |
| *Molossops greenhalli* | 15.9 | 35.5 | 12.6 |  |  | Insectivore |
| *Molossops mattogrossensis* | 7.5 | 28.5 | 9.2 |  |  | Insectivore |
| *Molossops neglectus* | 6.9 | 35.5 | 5.5 |  |  | Insectivore |
| *Molossops planirostris* | 12.8 | 31.5 | 12.9 |  |  | Insectivore |
| *Molossops temminckii* | 5.9 | 28.5 | 7.2 |  |  | Insectivore |
| *Molossus bondae* | 17.8 | 41.0 | 10.6 |  |  | Insectivore |
| *Molossus molossus* | 13.7 | 38.2 | 9.4 | 59.8 | 3.8 | Insectivore |
| *Molossus sinaloae* | 21.1 | 47.1 | 9.5 |  |  | Insectivore |
| *Monophyllus plethodon* | 15.3 | 43.5 | 8.1 |  |  | Frugivore |
| *Monophyllus redmani* | 8.8 | 39.4 | 5.7 |  |  | Frugivore |
| *Mops brachypterus* | 16.0 | 36.5 | 12.0 |  |  | Insectivore |
| *Mops condylurus* | 26.6 | 47.2 | 11.9 |  |  | Insectivore |
| *Mops congicus* | 42.8 | 55.5 | 13.9 |  |  | Insectivore |
| *Mops demonstrator* | 9.7 | 40.0 | 6.1 |  |  | Insectivore |
| *Mops midas* | 45.5 | 61.5 | 12.0 |  |  | Insectivore |
| *Mops mops* | 31.1 | 45.5 | 15.0 |  |  | Insectivore |
| *Mops nanulus* | 3.9 | 29.0 | 4.6 |  |  | Insectivore |
| *Mops petersoni* | 5.9 | 33.5 | 5.2 |  |  | Insectivore |
| *Mops sarasinorum* | 11.2 | 42.0 | 6.3 |  |  | Insectivore |
| *Mops spurrelli* | 8.1 | 27.5 | 10.7 |  |  | Insectivore |
| *Mops thersites* | 22.0 | 39.5 | 14.1 |  |  | Insectivore |
| *Mops trevori* | 21.2 | 52.5 | 7.7 |  |  | Insectivore |
| *Mormoops blainvilli* | 8.7 | 46.5 | 4.0 |  |  | Insectivore |
| *Mormoops megalophylla* | 16.1 | 55.0 | 5.3 | 66.7 | 3.6 | Insectivore |
| *Mormopterus acetabulosus* | 9.1 | 39.0 | 6.0 |  |  | Insectivore |
| *Mormopterus beccarii* | 14.3 | 36.0 | 11.0 |  |  | Insectivore |
| *Mormopterus doriae* | 8.4 | 38.0 | 5.8 |  |  | Insectivore |
| *Mormopterus jugularis* | 11.6 | 37.4 | 8.3 |  |  | Insectivore |
| *Mormopterus kalinowskii* | 7.5 | 36.5 | 5.6 |  |  | Insectivore |
| *Mormopterus minutus* | 4.3 | 30.0 | 4.7 |  |  | Insectivore |
| *Mormopterus norfolkensis* | 8.0 | 36.5 | 6.0 |  |  | Insectivore |
| *Mormopterus petrophilus* | 14.3 | 39.0 | 9.4 |  |  | Insectivore |
| *Mormopterus phrudus* | 6.1 | 34.0 | 5.3 |  |  | Insectivore |
| *Mormopterus planiceps* | 9.2 | 33.6 | 8.2 |  |  | Insectivore |
| *Mormopterus setiger* | 5.4 | 32.5 | 5.1 |  |  | Insectivore |
| *Mosia nigrescens* | 3.3 | 34.5 | 2.8 |  |  | Insectivore |
| *Murina aenea* | 7.5 | 35.5 | 6.0 |  |  | Insectivore |
| *Murina aurata* | 4.3 | 30.0 | 4.7 |  |  | Insectivore |
| *Murina cyclotis* | 9.4 | 34.4 | 7.9 |  |  | Insectivore |
| *Murina florium* | 4.4 | 34.5 | 3.7 |  |  | Insectivore |
| *Murina grisea* | 5.6 | 33.0 | 5.2 |  |  | Frugivore |
| *Murina huttoni* | 7.6 | 33.5 | 6.7 |  |  | Insectivore |
| *Murina leucogaster* | 7.5 | 42.0 | 4.3 |  |  | Insectivore |
| *Murina puta* | 6.9 | 35.5 | 5.5 |  |  | Insectivore |
| *Murina rozendaali* | 5.6 | 33.0 | 5.2 |  |  | Insectivore |
| *Murina silvatica* | 4.5 | 30.5 | 4.8 |  |  | Insectivore |
| *Murina suilla* | 4.0 | 30.0 | 4.4 |  |  | Insectivore |
| *Murina tenebrosa* | 6.4 | 34.5 | 5.4 |  |  | Insectivore |
| *Murina tubinaris* | 5.5 | 32.0 | 5.3 |  |  | Insectivore |
| *Murina ussuriensis* | 4.7 | 31.0 | 4.9 |  |  | Insectivore |
| *Musonycteris harrisoni* | 10.8 | 41.5 | 6.3 |  |  | Insectivore |
| *Myonycteris brachycephala* | 35.8 | 63.0 | 9.0 |  |  | Frugivore |
| *Myonycteris relicta* | 53.3 | 69.7 | 11.0 |  |  | Frugivore |
| *Myonycteris torquata* | 44.9 | 60.9 | 12.1 | 99.4 | 4.5 | Frugivore |
| *Myopterus daubentonii* | 18.5 | 50.0 | 7.4 |  |  | Insectivore |
| *Myopterus whitleyi* | 11.6 | 35.0 | 9.4 |  |  | Insectivore |
| *Myotis abei* | 6.1 | 34.0 | 5.3 |  |  | Insectivore |
| *Myotis adversus* | 10.4 | 39.8 | 6.6 |  |  | Insectivore |
| *Myotis aelleni* | 9.4 | 39.5 | 6.0 |  |  | Insectivore |
| *Myotis albescens* | 5.7 | 36.0 | 4.4 |  |  | Insectivore |
| *Myotis altarium* | 11.0 | 45.0 | 5.4 |  |  | Insectivore |
| *Myotis annectans* | 9.8 | 45.0 | 4.8 |  |  | Insectivore |
| *Myotis australis* | 7.8 | 37.0 | 5.7 |  |  | Insectivore |
| *Myotis austroriparius* | 7.4 | 38.0 | 5.1 |  |  | Insectivore |
| *Myotis bechsteini* | 9.5 | 42.0 | 5.4 |  |  | Insectivore |
| *Myotis blythi* | 23.8 | 58.5 | 7.0 |  |  | Insectivore |
| *Myotis bocagei* | 7.9 | 38.0 | 5.5 |  |  | Insectivore |
| *Myotis bombinus* | 9.7 | 40.0 | 6.1 |  |  | Insectivore |
| *Myotis brandti* | 5.3 | 35.0 | 4.3 |  |  | Insectivore |
| *Myotis californicus* | 4.4 | 33.1 | 4.0 |  |  | Insectivore |
| *Myotis capaccinii* | 8.2 | 40.0 | 5.1 |  |  | Insectivore |
| *Myotis chiloensis* | 8.4 | 38.0 | 5.8 |  |  | Insectivore |
| *Myotis chinensis* | 42.0 | 65.0 | 9.9 |  |  | Insectivore |
| *Myotis cobanensis* | 10.8 | 41.5 | 6.3 |  |  | Insectivore |
| *Myotis dasycneme* | 15.2 | 45.5 | 7.3 |  |  | Insectivore |
| *Myotis daubentoni* | 7.6 | 36.6 | 5.7 | 44.4 | 3.9 | Insectivore |
| *Myotis dominicensis* | 6.1 | 34.0 | 5.3 |  |  | Insectivore |
| *Myotis elegans* | 4.2 | 33.0 | 3.9 |  |  | Insectivore |
| *Myotis emarginatus* | 7.6 | 40.0 | 4.7 |  |  | Insectivore |
| *Myotis evotis* | 6.9 | 36.7 | 5.1 |  |  | Insectivore |
| *Myotis findleyi* | 4.9 | 31.5 | 4.9 |  |  | Insectivore |
| *Myotis formosus* | 7.1 | 49.0 | 2.9 |  |  | Insectivore |
| *Myotis fortidens* | 4.4 | 37.0 | 3.2 |  |  | Insectivore |
| *Myotis frater* | 7.5 | 38.5 | 5.1 |  |  | Insectivore |
| *Myotis goudoti* | 5.6 | 39.5 | 3.6 |  |  | Insectivore |
| *Myotis grisescens* | 10.8 | 43.3 | 5.8 |  |  | Insectivore |
| *Myotis hasseltii* | 8.7 | 38.5 | 5.9 |  |  | Insectivore |
| *Myotis horsfieldii* | 6.1 | 38.3 | 4.1 |  |  | Insectivore |
| *Myotis hosonoi* | 6.6 | 35.0 | 5.4 |  |  | Insectivore |
| *Myotis ikonnikovi* | 5.9 | 33.5 | 5.2 |  |  | Insectivore |
| *Myotis insularum* | 6.1 | 34.0 | 5.3 |  |  | Insectivore |
| *Myotis keaysi* | 5.5 | 36.5 | 4.1 |  |  | Insectivore |
| *Myotis keenii* | 6.5 | 36.5 | 4.9 |  |  | Insectivore |
| *Myotis leibii* | 5.2 | 32.5 | 4.9 |  |  | Insectivore |
| *Myotis levis* | 5.5 | 38.0 | 3.8 |  |  | Insectivore |
| *Myotis longipes* | 7.2 | 36.0 | 5.6 |  |  | Insectivore |
| *Myotis lucifugus* | 8.8 | 37.8 | 6.2 | 52.7 | 3.2 | Insectivore |
| *Myotis macrodactylus* | 7.5 | 38.4 | 5.1 |  |  | Insectivore |
| *Myotis macrotarsus* | 12.6 | 47.0 | 5.7 |  |  | Insectivore |
| *Myotis martiniquensis* | 7.5 | 36.5 | 5.6 |  |  | Insectivore |
| *Myotis montivagus* | 8.3 | 43.0 | 4.5 |  |  | Insectivore |
| *Myotis morrisi* | 14.1 | 45.5 | 6.8 |  |  | Insectivore |
| *Myotis muricola* | 4.8 | 34.7 | 4.0 |  |  | Insectivore |
| *Myotis myotis* | 25.6 | 63.9 | 6.3 | 72.0 | 4.9 | Insectivore |
| *Myotis nattereri* | 7.3 | 39.4 | 4.7 |  |  | Insectivore |
| *Myotis nesopolus* | 3.6 | 31.5 | 3.6 |  |  | Insectivore |
| *Myotis oreias* | 8.4 | 38.0 | 5.8 |  |  | Insectivore |
| *Myotis oxyotus* | 5.7 | 40.0 | 3.5 |  |  | Insectivore |
| *Myotis ozensis* | 5.9 | 33.5 | 5.2 |  |  | Insectivore |
| *Myotis peninsularis* | 9.1 | 39.0 | 6.0 |  |  | Insectivore |
| *Myotis pequinius* | 17.4 | 49.0 | 7.3 |  |  | Insectivore |
| *Myotis planiceps* | 3.0 | 26.5 | 4.3 |  |  | Insectivore |
| *Myotis pruinosus* | 4.9 | 31.5 | 4.9 |  |  | Insectivore |
| *Myotis ricketti* | 26.2 | 56.5 | 8.2 |  |  | Carnivore |
| *Myotis ridleyi* | 4.1 | 30.0 | 4.5 |  |  | Insectivore |
| *Myotis riparius* | 4.6 | 35.0 | 3.7 |  |  | Insectivore |
| *Myotis rosseti* | 3.3 | 29.0 | 3.9 |  |  | Insectivore |
| *Myotis ruber* | 5.0 | 40.0 | 3.1 |  |  | Insectivore |
| *Myotis schaubi* | 12.0 | 43.0 | 6.5 |  |  | Insectivore |
| *Myotis scotti* | 8.7 | 38.5 | 5.9 |  |  | Insectivore |
| *Myotis sicarius* | 20.1 | 51.5 | 7.6 |  |  | Insectivore |
| *Myotis siligorensis* | 2.9 | 33.0 | 2.7 |  |  | Insectivore |
| *Myotis simus* | 8.1 | 37.5 | 5.8 |  |  | Insectivore |
| *Myotis sodalis* | 7.2 | 38.5 | 4.8 | 47.4 | 3.2 | Insectivore |
| *Myotis stalkeri* | 16.4 | 48.0 | 7.1 |  |  | Insectivore |
| *Myotis thysanodes* | 8.5 | 43.4 | 4.5 | 52.4 | 3.1 | Insectivore |
| *Myotis tricolor* | 13.7 | 49.5 | 5.6 |  |  | Insectivore |
| *Myotis velifer* | 9.8 | 42.0 | 5.6 |  |  | Insectivore |
| *Myotis vivesi* | 25.6 | 61.0 | 6.9 |  |  | Insectivore |
| *Myotis volans* | 8.7 | 37.5 | 6.2 |  |  | Insectivore |
| *Myotis welwitschii* | 15.9 | 57.8 | 4.8 |  |  | Insectivore |
| *Myotis yesoensis* | 6.1 | 34.0 | 5.3 |  |  | Insectivore |
| *Myotis yumanensis* | 5.2 | 35.1 | 4.2 |  |  | Insectivore |
| *Mystacina robusta* | 27.4 | 44.2 | 14.0 |  |  | Omnivore |
| *Mystacina tuberculata* | 13.1 | 42.5 | 7.3 |  |  | Omnivore |
| *Myzopoda aurita* | 9.1 | 48.0 | 3.9 | 58.0 | 2.7 | Insectivore |
| *Nanonycteris veldkampii* | 21.9 | 48.0 | 9.5 |  |  | Frugivore |
| *Natalus lepidus* | 3.9 | 29.0 | 4.6 |  |  | Insectivore |
| *Natalus micropus* | 5.9 | 33.5 | 5.2 |  |  | Insectivore |
| *Natalus stramineus* | 5.7 | 38.9 | 3.8 |  |  | Insectivore |
| *Natalus tumidifrons* | 3.6 | 33.0 | 3.3 |  |  | Insectivore |
| *Natalus tumidirostris* | 6.3 | 38.5 | 4.3 |  |  | Insectivore |
| *Neopteryx frosti* | 176.7 | 110.0 | 14.6 |  |  | Frugivore |
| *Noctilio albiventris* | 31.5 | 60.0 | 8.7 | 66.5 | 7.1 | Insectivore |
| *Notopteris macdonaldi* | 68.2 | 66.0 | 15.7 |  |  | Frugivore |
| *Notopteris macdonaldi* | 68.2 | 66.0 | 15.6 |  |  | Frugivore |
| *Nyctalus aviator* | 31.9 | 60.5 | 8.7 |  |  | Carnivore |
| *Nyctalus azoreum* | 8.8 | 38.6 | 5.9 |  |  | Insectivore |
| *Nyctalus lasiopterus* | 46.0 | 64.1 | 11.2 |  |  | Carnivore |
| *Nyctalus leisleri* | 12.5 | 43.2 | 6.7 |  |  | Insectivore |
| *Nyctalus noctula* | 28.5 | 51.6 | 10.7 | 75.0 | 5.1 | Insectivore |
| *Nycteris arge* | 10.8 | 41.5 | 6.3 |  |  | Insectivore |
| *Nycteris gambiensis* | 7.1 | 39.5 | 4.6 |  |  | Insectivore |
| *Nycteris grandis* | 29.8 | 58.5 | 8.7 | 76.0 | 5.2 | Carnivore |
| *Nycteris hispida* | 8.0 | 39.0 | 5.2 |  |  | Insectivore |
| *Nycteris intermedia* | 6.9 | 35.5 | 5.5 |  |  | Insectivore |
| *Nycteris javanica* | 17.8 | 46.0 | 8.4 |  |  | Insectivore |
| *Nycteris macrotis* | 14.5 | 47.5 | 6.4 |  |  | Insectivore |
| *Nycteris major* | 15.5 | 47.0 | 7.0 |  |  | Insectivore |
| *Nycteris nana* | 7.0 | 34.0 | 6.0 |  |  | Insectivore |
| *Nycteris thebaica* | 9.2 | 45.3 | 4.5 | 52.7 | 3.3 | Insectivore |
| *Nycteris tragata* | 14.4 | 49.5 | 5.9 |  |  | Insectivore |
| *Nycteris woodi* | 7.6 | 39.5 | 4.9 |  |  | Insectivore |
| *Nycticeius balstoni* | 11.9 | 35.5 | 9.5 |  |  | Insectivore |
| *Nycticeius greyii* | 10.0 | 30.0 | 11.1 |  |  | Insectivore |
| *Nycticeius humeralis* | 9.1 | 33.7 | 8.0 |  |  | Insectivore |
| *Nycticeius rueppellii* | 26.4 | 53.0 | 9.4 |  |  | Insectivore |
| *Nycticeius sanborni* | 8.1 | 33.0 | 7.5 |  |  | Insectivore |
| *Nycticeius schlieffeni* | 5.1 | 31.0 | 5.3 |  |  | Insectivore |
| *Nyctimene aello* | 85.3 | 82.7 | 12.5 | 122.0 | 5.7 | Frugivore |
| *Nyctimene albiventer* | 30.0 | 54.8 | 10.0 | 82.3 | 4.4 | Frugivore |
| *Nyctimene cephalotes* | 44.9 | 65.0 | 10.6 | 94.7 | 5.0 | Frugivore |
| *Nyctimene certans* | 43.2 | 60.7 | 11.7 | 86.4 | 5.8 | Frugivore |
| *Nyctimene cyclotis* | 48.8 | 54.2 | 16.6 |  |  | Frugivore |
| *Nyctimene draconilla* | 30.2 | 50.0 | 12.1 | 110.0 | 2.5 | Frugivore |
| *Nyctimene major* | 107.1 | 76.1 | 18.5 | 134.0 | 6.0 | Frugivore |
| *Nyctimene malaitensis* | 78.4 | 65.1 | 18.5 | 119.7 | 5.5 | Frugivore |
| *Nyctimene masalai* | 53.2 | 66.7 | 12.0 |  |  | Frugivore |
| *Nyctimene minutus* | 21.3 | 52.6 | 7.7 |  |  | Frugivore |
| *Nyctimene rabori* | 68.3 | 77.0 | 11.5 |  |  | Frugivore |
| *Nyctimene robinsoni* | 48.8 | 65.4 | 11.4 |  |  | Frugivore |
| *Nyctimene sanctacrucis* | 59.3 | 75.1 | 10.5 |  |  | Frugivore |
| *Nyctimene vizcaccia* | 42.0 | 58.1 | 12.4 | 93.9 | 4.8 | Frugivore |
| *Nyctinomops aurispinosus* | 18.4 | 49.5 | 7.5 |  |  | Insectivore |
| *Nyctinomops femorosaccus* | 15.0 | 47.5 | 6.7 |  |  | Insectivore |
| *Nyctinomops laticaudatus* | 13.1 | 43.2 | 7.0 |  |  | Insectivore |
| *Nyctinomops macrotis* | 16.4 | 61.0 | 4.4 |  |  | Insectivore |
| *Nyctophilus arnhemensis* | 6.8 | 37.5 | 4.9 |  |  | Insectivore |
| *Nyctophilus bifax* | 9.9 | 43.5 | 5.2 |  |  | Insectivore |
| *Nyctophilus gouldi* | 11.3 | 39.7 | 7.2 |  |  | Insectivore |
| *Nyctophilus microdon* | 9.1 | 39.0 | 6.0 |  |  | Insectivore |
| *Nyctophilus microtis* | 7.3 | 39.0 | 4.8 |  |  | Insectivore |
| *Nyctophilus timoriensis* | 11.0 | 43.4 | 5.9 |  |  | Insectivore |
| *Nyctophilus walkeri* | 4.4 | 33.0 | 4.1 |  |  | Insectivore |
| *Otomops formosus* | 30.4 | 59.5 | 8.6 |  |  | Insectivore |
| *Otomops martiensseni* | 34.9 | 65.3 | 8.2 |  |  | Insectivore |
| *Otomops papuensis* | 18.5 | 50.0 | 7.4 |  |  | Insectivore |
| *Otomops secundus* | 27.6 | 57.5 | 8.3 |  |  | Insectivore |
| *Otomops wroughtoni* | 39.2 | 65.0 | 9.3 |  |  | Insectivore |
| *Otonycteris hemprichii* | 22.0 | 63.1 | 5.5 | 64.6 | 5.3 | Insectivore |
| *Otopteropus cartilagonodus* | 16.9 | 47.0 | 7.7 |  |  | Frugivore |
| *Paracoelops megalotis* | 11.2 | 42.0 | 6.3 |  |  | Insectivore |
| *Paranyctimene raptor* | 24.9 | 51.7 | 9.3 | 77.7 | 4.1 | Frugivore |
| *Penthetor lucasi* | 35.4 | 60.5 | 9.7 |  |  | Frugivore |
| *Phylloderma stenops* | 55.8 | 71.9 | 10.8 |  |  | Omnivore |
| *Phyllonycteris aphylla* | 14.2 | 46.5 | 6.5 | 70.0 | 2.9 | Omnivore |
| *Phyllonycteris poeyi* | 15.6 | 47.8 | 6.8 | 64.0 | 3.8 | Omnivore |
| *Phyllops falcatus* | 10.8 | 41.5 | 6.3 |  |  | Frugivore |
| *Phyllostomus discolor* | 36.7 | 62.5 | 9.4 |  |  | Omnivore |
| *Phyllostomus elongatus* | 41.8 | 66.0 | 9.6 |  |  | Omnivore |
| *Phyllostomus hastatus* | 91.4 | 83.3 | 13.2 | 107.0 | 8.0 | Omnivore |
| *Phyllostomus latifolius* | 134.0 | 58.0 | 39.8 |  |  | Omnivore |
| *Pipistrellus abramus* | 5.9 | 33.9 | 5.1 |  |  | Insectivore |
| *Pipistrellus aegyptius* | 4.7 | 31.0 | 4.9 |  |  | Insectivore |
| *Pipistrellus aero* | 5.1 | 32.0 | 5.0 |  |  | Insectivore |
| *Pipistrellus anthonyi* | 8.4 | 38.0 | 5.8 |  |  | Insectivore |
| *Pipistrellus arabicus* | 3.2 | 30.5 | 3.4 |  |  | Insectivore |
| *Pipistrellus ariel* | 4.1 | 30.0 | 4.5 |  |  | Insectivore |
| *Pipistrellus cadornae* | 6.7 | 35.0 | 5.5 |  |  | Insectivore |
| *Pipistrellus ceylonicus* | 8.1 | 41.5 | 4.7 |  |  | Insectivore |
| *Pipistrellus circumdatus* | 10.4 | 40.5 | 6.3 |  |  | Insectivore |
| *Pipistrellus coromandra* | 4.6 | 30.5 | 4.9 |  |  | Insectivore |
| *Pipistrellus crassulus* | 6.1 | 28.5 | 7.5 |  |  | Insectivore |
| *Pipistrellus cuprosus* | 6.9 | 35.5 | 5.5 |  |  | Insectivore |
| *Pipistrellus dormeri* | 6.8 | 34.5 | 5.7 |  |  | Insectivore |
| *Pipistrellus eisentrauti* | 6.1 | 33.0 | 5.6 |  |  | Insectivore |
| *Pipistrellus endoi* | 5.1 | 32.0 | 5.0 |  |  | Insectivore |
| *Pipistrellus hesperus* | 3.6 | 30.5 | 3.8 |  |  | Insectivore |
| *Pipistrellus imbricatus* | 6.3 | 34.3 | 5.3 |  |  | Insectivore |
| *Pipistrellus inexspectatus* | 4.9 | 31.5 | 4.9 |  |  | Insectivore |
| *Pipistrellus javanicus* | 4.9 | 32.7 | 4.6 |  |  | Insectivore |
| *Pipistrellus joffrei* | 9.1 | 39.0 | 6.0 |  |  | Insectivore |
| *Pipistrellus kitcheneri* | 7.2 | 36.0 | 5.6 |  |  | Insectivore |
| *Pipistrellus kuhlii* | 6.1 | 34.7 | 5.0 | 46.0 | 2.9 | Insectivore |
| *Pipistrellus lophurus* | 6.6 | 35.0 | 5.4 |  |  | Insectivore |
| *Pipistrellus macrotis* | 5.1 | 32.0 | 5.0 |  |  | Insectivore |
| *Pipistrellus maderensis* | 4.9 | 31.5 | 4.9 |  |  | Insectivore |
| *Pipistrellus minahassae* | 7.2 | 36.0 | 5.6 |  |  | Insectivore |
| *Pipistrellus mordax* | 10.5 | 41.0 | 6.2 |  |  | Insectivore |
| *Pipistrellus musciculus* | 2.0 | 23.0 | 3.8 |  |  | Insectivore |
| *Pipistrellus nanulus* | 2.5 | 28.0 | 3.2 |  |  | Insectivore |
| *Pipistrellus nanus* | 3.9 | 29.0 | 4.6 |  |  | Insectivore |
| *Pipistrellus nathusii* | 7.4 | 34.0 | 6.4 |  |  | Insectivore |
| *Pipistrellus paterculus* | 4.9 | 31.5 | 4.9 |  |  | Insectivore |
| *Pipistrellus permixtus* | 5.9 | 33.5 | 5.2 |  |  | Insectivore |
| *Pipistrellus petersi* | 9.1 | 39.0 | 6.0 |  |  | Insectivore |
| *Pipistrellus pipistrellus* | 5.3 | 31.5 | 5.3 | 40.0 | 3.3 | Insectivore |
| *Pipistrellus pulveratus* | 5.5 | 34.0 | 4.8 |  |  | Insectivore |
| *Pipistrellus rueppelli* | 7.1 | 32.6 | 6.7 | 45.0 | 3.5 | Insectivore |
| *Pipistrellus rusticus* | 4.6 | 28.0 | 5.8 |  |  | Insectivore |
| *Pipistrellus savii* | 6.3 | 34.5 | 5.3 |  |  | Insectivore |
| *Pipistrellus societatis* | 8.7 | 38.5 | 5.9 |  |  | Insectivore |
| *Pipistrellus stenopterus* | 15.7 | 40.0 | 9.8 |  |  | Insectivore |
| *Pipistrellus sturdeei* | 4.3 | 30.0 | 4.7 |  |  | Insectivore |
| *Pipistrellus subflavus* | 5.7 | 34.0 | 5.0 |  |  | Insectivore |
| *Pipistrellus tasmaniensis* | 22.5 | 50.0 | 9.0 |  |  | Insectivore |
| *Pipistrellus tenuis* | 3.5 | 29.9 | 3.9 |  |  | Insectivore |
| *Platalina genovensium* | 16.4 | 48.0 | 7.1 |  |  | Frugivore |
| *Platyrrhinus aurarius* | 35.1 | 51.5 | 13.2 |  |  | Frugivore |
| *Platyrrhinus brachycephalus* | 14.3 | 39.0 | 9.4 |  |  | Frugivore |
| *Platyrrhinus dorsalis* | 26.0 | 47.5 | 11.5 |  |  | Frugivore |
| *Platyrrhinus helleri* | 13.4 | 38.1 | 9.3 |  |  | Frugivore |
| *Platyrrhinus infuscus* | 50.8 | 56.5 | 15.9 |  |  | Frugivore |
| *Platyrrhinus lineatus* | 24.3 | 46.6 | 11.2 |  |  | Frugivore |
| *Platyrrhinus recifinus* | 10.8 | 41.5 | 6.3 |  |  | Frugivore |
| *Platyrrhinus umbratus* | 25.2 | 45.5 | 12.2 |  |  | Frugivore |
| *Platyrrhinus vittatus* | 37.0 | 57.1 | 11.4 | 82.0 | 5.5 | Frugivore |
| *Plecotus auritus* | 8.2 | 37.7 | 5.8 |  |  | Insectivore |
| *Plecotus austriacus* | 6.8 | 39.7 | 4.3 | 41.0 | 4.0 | Insectivore |
| *Plecotus rafinesquii* | 9.2 | 43.0 | 4.9 |  |  | Insectivore |
| *Plecotus townsendii* | 10.3 | 43.4 | 5.5 | 57.5 | 3.1 | Insectivore |
| *Plerotes anchietae* | 20.0 | 51.4 | 7.6 |  |  | Frugivore |
| *Promops centralis* | 29.8 | 53.0 | 10.6 |  |  | Insectivore |
| *Promops nasutus* | 15.5 | 47.3 | 6.9 |  |  | Insectivore |
| *Ptenochirus jagori* | 79.2 | 84.3 | 11.1 |  |  | Frugivore |
| *Ptenochirus minor* | 47.0 | 69.5 | 9.7 |  |  | Frugivore |
| *Pteralopex anceps* | 574.8 | 165.5 | 21.0 | 262.3 | 8.4 | Frugivore |
| *Pteralopex atrata* | 490.8 | 142.5 | 24.2 | 207.4 | 11.4 | Frugivore |
| *Pteronotus davyi* | 9.5 | 45.1 | 4.7 |  |  | Insectivore |
| *Pteronotus gymnonotus* | 13.6 | 51.7 | 5.1 |  |  | Insectivore |
| *Pteronotus macleayii* | 12.4 | 43.5 | 6.5 |  |  | Insectivore |
| *Pteronotus parnellii* | 19.6 | 60.1 | 5.4 | 57.0 | 6.0 | Insectivore |
| *Pteronotus personatus* | 8.0 | 44.5 | 4.0 | 45.2 | 3.9 | Insectivore |
| *Pteronotus quadridens* | 5.6 | 37.5 | 4.0 |  |  | Insectivore |
| *Pteropus admiralitatum* | 306.5 | 115.0 | 23.2 | 180.0 | 9.5 | Frugivore |
| *Pteropus aldabrensis* | 310.3 | 136.1 | 16.8 |  |  | Frugivore |
| *Pteropus alecto* | 610.1 | 166.0 | 22.1 | 254.6 | 9.4 | Frugivore |
| *Pteropus anetianus* | 297.2 | 121.5 | 20.1 |  |  | Frugivore |
| *Pteropus brunneus* | 200.0 | 118.0 | 14.4 | 117.6 | 14.5 | Frugivore |
| *Pteropus caniceps* | 525.1 | 140.2 | 26.7 | 216.7 | 11.2 | Frugivore |
| *Pteropus chrysoproctus* | 730.2 | 170.0 | 25.3 | 253.6 | 11.4 | Frugivore |
| *Pteropus conspicillatus* | 760.7 | 174.1 | 25.1 |  |  | Frugivore |
| *Pteropus dasymallus* | 491.9 | 131.0 | 28.7 |  |  | Frugivore |
| *Pteropus faunulus* | 222.6 | 114.7 | 16.9 | 169.5 | 7.7 | Frugivore |
| *Pteropus fundatus* | 210.7 | 101.6 | 20.4 | 147.4 | 9.7 | Frugivore |
| *Pteropus giganteus* | 824.9 | 178.0 | 26.0 |  |  | Frugivore |
| *Pteropus gilliardorum* | 406.9 | 114.6 | 31.0 | 159.0 | 16.1 | Frugivore |
| *Pteropus howensis* | 232.9 | 119.0 | 16.4 | 184.6 | 6.8 | Frugivore |
| *Pteropus hypomelanus* | 435.6 | 132.0 | 25.0 | 204.9 | 10.4 | Frugivore |
| *Pteropus insularis* | 155.2 | 105.2 | 14.0 |  |  | Frugivore |
| *Pteropus intermedius* | 735.5 | 181.0 | 22.4 |  |  | Frugivore |
| *Pteropus livingstonii* | 734.3 | 172.9 | 24.6 | 340.0 | 6.4 | Frugivore |
| *Pteropus lombocensis* | 256.2 | 113.2 | 20.0 |  |  | Frugivore |
| *Pteropus lylei* | 319.8 | 151.7 | 13.9 | 237.5 | 5.7 | Frugivore |
| *Pteropus macrotis* | 366.0 | 135.2 | 20.0 | 211.2 | 8.2 | Frugivore |
| *Pteropus mahaganus* | 297.3 | 137.5 | 15.7 | 189.9 | 8.2 | Frugivore |
| *Pteropus mariannus* | 458.6 | 135.5 | 25.0 | 217.5 | 9.7 | Frugivore |
| *Pteropus melanopogon* | 874.9 | 191.5 | 23.9 | 285.0 | 10.8 | Frugivore |
| *Pteropus melanotus* | 418.1 | 148.6 | 18.9 |  |  | Frugivore |
| *Pteropus molossinus* | 121.4 | 96.5 | 13.0 |  |  | Frugivore |
| *Pteropus neohibernicus* | 1020.0 | 195.1 | 26.8 | 279.1 | 13.1 | Frugivore |
| *Pteropus niger* | 474.4 | 160.4 | 18.4 |  |  | Frugivore |
| *Pteropus nitendiensis* | 275.6 | 119.3 | 19.4 | 175.5 | 8.9 | Frugivore |
| *Pteropus ocularis* | 228.8 | 138.7 | 11.9 | 202.9 | 5.6 | Frugivore |
| *Pteropus ornatus* | 336.3 | 154.0 | 14.2 | 183.2 | 10.0 | Frugivore |
| *Pteropus pelewensis* | 193.8 | 113.6 | 15.0 |  |  | Frugivore |
| *Pteropus personatus* | 130.8 | 91.9 | 15.5 | 136.4 | 7.0 | Frugivore |
| *Pteropus pilosus* | 441.1 | 151.4 | 19.2 |  |  | Frugivore |
| *Pteropus pohlei* | 352.8 | 131.0 | 20.6 | 197.3 | 9.1 | Frugivore |
| *Pteropus poliocephalus* | 702.8 | 162.0 | 26.8 | 272.6 | 9.5 | Frugivore |
| *Pteropus pselaphon* | 321.5 | 135.6 | 17.5 |  |  | Frugivore |
| *Pteropus pumilus* | 184.1 | 108.0 | 15.8 |  |  | Frugivore |
| *Pteropus rayneri* | 661.3 | 158.6 | 26.3 | 255.5 | 10.1 | Frugivore |
| *Pteropus rodricensis* | 256.2 | 125.3 | 16.3 |  |  | Frugivore |
| *Pteropus rufus* | 535.3 | 162.0 | 20.4 |  |  | Frugivore |
| *Pteropus samoensis* | 310.0 | 138.4 | 16.2 |  |  | Frugivore |
| *Pteropus scapulatus* | 380.4 | 131.5 | 22.0 |  |  | Frugivore |
| *Pteropus seychellensis* | 492.2 | 153.2 | 21.0 |  |  | Frugivore |
| *Pteropus speciosus* | 234.9 | 121.5 | 15.9 |  |  | Frugivore |
| *Pteropus subniger* | 127.2 | 98.1 | 13.2 |  |  | Frugivore |
| *Pteropus tokudae* | 153.7 | 93.5 | 17.6 | 145.5 | 7.3 | Frugivore |
| *Pteropus tonganus* | 561.5 | 136.2 | 30.3 | 177.3 | 17.9 | Frugivore |
| *Pteropus tuberculatus* | 226.6 | 120.0 | 15.7 |  |  | Frugivore |
| *Pteropus vampyrus* | 1030.0 | 200.0 | 25.8 |  |  | Frugivore |
| *Pteropus vetulus* | 151.5 | 101.8 | 14.6 | 136.0 | 8.2 | Frugivore |
| *Pteropus voeltzkowi* | 542.4 | 156.0 | 22.3 |  |  | Frugivore |
| *Pteropus woodfordi* | 122.7 | 88.3 | 15.7 | 141.5 | 6.1 | Frugivore |
| *Pteropus yapensis* | 289.6 | 130.7 | 17.0 |  |  | Frugivore |
| *Pygoderma bilabiatum* | 18.5 | 40.0 | 11.6 |  |  | Frugivore |
| *Rhinolophus acuminatus* | 12.1 | 49.6 | 4.9 |  |  | Insectivore |
| *Rhinolophus adami* | 17.4 | 49.0 | 7.3 |  |  | Insectivore |
| *Rhinolophus affinis* | 13.7 | 52.2 | 5.0 |  |  | Insectivore |
| *Rhinolophus alcyone* | 18.6 | 51.5 | 7.0 |  |  | Insectivore |
| *Rhinolophus arcuatus* | 9.0 | 45.7 | 4.3 |  |  | Insectivore |
| *Rhinolophus beddomei* | 29.8 | 59.1 | 8.5 |  |  | Insectivore |
| *Rhinolophus blasii* | 10.3 | 44.5 | 5.2 |  |  | Insectivore |
| *Rhinolophus borneensis* | 12.8 | 44.0 | 6.6 |  |  | Insectivore |
| *Rhinolophus canuti* | 17.9 | 49.5 | 7.3 |  |  | Insectivore |
| *Rhinolophus capensis* | 12.9 | 49.5 | 5.3 |  |  | Insectivore |
| *Rhinolophus celebensis* | 10.8 | 41.5 | 6.3 |  |  | Insectivore |
| *Rhinolophus coelophyllus* | 7.1 | 43.0 | 3.8 |  |  | Insectivore |
| *Rhinolophus cognatus* | 9.4 | 39.5 | 6.0 |  |  | Insectivore |
| *Rhinolophus cornutus* | 7.3 | 40.5 | 4.4 |  |  | Insectivore |
| *Rhinolophus creaghi* | 16.9 | 48.5 | 7.2 |  |  | Insectivore |
| *Rhinolophus darlingi* | 8.9 | 47.5 | 4.0 |  |  | Insectivore |
| *Rhinolophus deckenii* | 20.1 | 51.5 | 7.6 |  |  | Insectivore |
| *Rhinolophus denti* | 6.3 | 40.0 | 3.9 |  |  | Insectivore |
| *Rhinolophus eloquens* | 19.2 | 57.0 | 5.9 |  |  | Insectivore |
| *Rhinolophus euryale* | 9.3 | 47.5 | 4.1 |  |  | Insectivore |
| *Rhinolophus euryotis* | 14.3 | 56.0 | 4.6 |  |  | Insectivore |
| *Rhinolophus ferrumequinum* | 22.6 | 63.7 | 5.6 |  |  | Insectivore |
| *Rhinolophus fumigatus* | 13.1 | 53.5 | 4.6 |  |  | Insectivore |
| *Rhinolophus guineensis* | 15.0 | 46.5 | 6.9 |  |  | Insectivore |
| *Rhinolophus hildebrandtii* | 26.0 | 64.4 | 6.3 |  |  | Insectivore |
| *Rhinolophus hilli* | 13.6 | 53.1 | 4.8 | 60.9 | 3.7 | Insectivore |
| *Rhinolophus hipposideros* | 4.6 | 37.5 | 3.2 | 39.0 | 3.0 | Insectivore |
| *Rhinolophus imaizumii* | 10.8 | 41.5 | 6.3 |  |  | Insectivore |
| *Rhinolophus inops* | 13.7 | 53.5 | 4.8 |  |  | Insectivore |
| *Rhinolophus keyensis* | 6.3 | 43.7 | 3.3 |  |  | Insectivore |
| *Rhinolophus landeri* | 9.4 | 42.0 | 5.3 |  |  | Insectivore |
| *Rhinolophus lepidus* | 5.5 | 40.0 | 3.4 |  |  | Insectivore |
| *Rhinolophus luctus* | 40.1 | 67.5 | 8.8 |  |  | Insectivore |
| *Rhinolophus maclandi* | 35.8 | 63.0 | 9.0 |  |  | Insectivore |
| *Rhinolophus maclaudi* | 35.8 | 63.0 | 9.0 |  |  | Insectivore |
| *Rhinolophus macrotis* | 6.2 | 42.0 | 3.5 |  |  | Insectivore |
| *Rhinolophus malayanus* | 6.7 | 41.5 | 3.9 |  |  | Insectivore |
| *Rhinolophus marshalli* | 5.0 | 45.5 | 2.4 |  |  | Insectivore |
| *Rhinolophus megaphyllus* | 10.2 | 47.2 | 4.6 |  |  | Insectivore |
| *Rhinolophus mehelyi* | 14.0 | 48.5 | 6.0 |  |  | Insectivore |
| *Rhinolophus mitratus* | 27.6 | 57.5 | 8.3 |  |  | Insectivore |
| *Rhinolophus monoceros* | 7.5 | 36.5 | 5.6 |  |  | Insectivore |
| *Rhinolophus nereis* | 13.7 | 45.0 | 6.7 |  |  | Insectivore |
| *Rhinolophus osgoodi* | 12.4 | 43.5 | 6.5 |  |  | Insectivore |
| *Rhinolophus paradoxolophus* | 8.2 | 54.0 | 2.8 |  |  | Insectivore |
| *Rhinolophus pearsonii* | 11.6 | 52.0 | 4.3 |  |  | Insectivore |
| *Rhinolophus philippinensis* | 10.9 | 51.0 | 4.2 |  |  | Insectivore |
| *Rhinolophus pusillus* | 5.2 | 37.6 | 3.6 |  |  | Insectivore |
| *Rhinolophus rex* | 32.6 | 61.0 | 8.8 |  |  | Insectivore |
| *Rhinolophus robinsoni* | 8.5 | 44.0 | 4.4 |  |  | Insectivore |
| *Rhinolophus rouxi* | 12.3 | 49.0 | 5.1 |  |  | Insectivore |
| *Rhinolophus rouxii* | 12.3 | 49.0 | 5.1 |  |  | Insectivore |
| *Rhinolophus ruwenzorii* | 25.5 | 56.0 | 8.1 |  |  | Insectivore |
| *Rhinolophus sedulus* | 8.7 | 46.0 | 4.1 |  |  | Insectivore |
| *Rhinolophus shameli* | 9.6 | 44.5 | 4.9 |  |  | Insectivore |
| *Rhinolophus silvestris* | 21.2 | 52.5 | 7.7 |  |  | Insectivore |
| *Rhinolophus simulator* | 8.1 | 44.5 | 4.1 |  |  | Insectivore |
| *Rhinolophus stheno* | 7.9 | 45.3 | 3.9 |  |  | Insectivore |
| *Rhinolophus subbadius* | 5.9 | 33.5 | 5.2 |  |  | Insectivore |
| *Rhinolophus subrufus* | 24.3 | 55.0 | 8.0 |  |  | Insectivore |
| *Rhinolophus swinnyi* | 7.1 | 42.0 | 4.0 |  |  | Insectivore |
| *Rhinolophus thomasi* | 8.3 | 48.5 | 3.5 |  |  | Insectivore |
| *Rhinolophus trifoliatus* | 15.2 | 50.5 | 5.9 |  |  | Insectivore |
| *Rhinolophus virgo* | 6.1 | 38.0 | 4.2 |  |  | Insectivore |
| *Rhinolophus yunanensis* | 19.3 | 61.0 | 5.2 |  |  | Insectivore |
| *Rhinolophus yunnanensis* | 19.3 | 61.0 | 5.2 |  |  | Insectivore |
| *Rhinonicteris aurantia* | 9.0 | 47.7 | 4.0 | 50.0 | 3.6 | Insectivore |
| *Rhinophylla alethina* | 6.9 | 35.5 | 5.5 |  |  | Frugivore |
| *Rhinophylla fischerae* | 9.6 | 32.0 | 9.4 |  |  | Frugivore |
| *Rhinophylla pumilio* | 9.6 | 33.5 | 8.5 |  |  | Frugivore |
| *Rhinopoma hardwickii* | 13.1 | 59.5 | 3.7 | 59.8 | 3.7 | Insectivore |
| *Rhinopoma microphyllum* | 28.0 | 68.1 | 6.0 | 61.8 | 7.3 | Insectivore |
| *Rhinopoma muscatellum* | 9.1 | 50.0 | 3.6 |  |  | Insectivore |
| *Rhogeessa alleni* | 5.4 | 32.5 | 5.1 |  |  | Insectivore |
| *Rhogeessa genowaysi* | 3.9 | 29.0 | 4.6 |  |  | Insectivore |
| *Rhogeessa gracilis* | 5.1 | 32.0 | 5.0 |  |  | Insectivore |
| *Rhogeessa minutilla* | 3.8 | 27.5 | 5.0 |  |  | Insectivore |
| *Rhogeessa mira* | 2.7 | 25.5 | 4.1 |  |  | Insectivore |
| *Rhogeessa parvula* | 4.4 | 29.0 | 5.2 |  |  | Insectivore |
| *Rhogeessa tumida* | 4.6 | 29.5 | 5.3 |  |  | Insectivore |
| *Rhynchonycteris naso* | 4.1 | 38.1 | 2.9 | 42.9 | 2.2 | Insectivore |
| *Rousettus aegyptiacus* | 134.0 | 93.2 | 15.4 | 167.4 | 4.8 | Frugivore |
| *Rousettus amplexicaudatus* | 74.4 | 76.9 | 12.6 | 107.6 | 6.4 | Frugivore |
| *Rousettus angolensis* | 68.3 | 73.1 | 12.8 | 105.2 | 6.2 | Frugivore |
| *Rousettus bidens* | 123.2 | 97.0 | 13.1 |  |  | Frugivore |
| *Rousettus celebensis* | 63.1 | 71.8 | 12.2 | 104.5 | 5.8 | Frugivore |
| *Rousettus lanosus* | 104.8 | 88.2 | 13.5 |  |  | Frugivore |
| *Rousettus leschenaulti* | 84.9 | 82.3 | 12.5 |  |  | Frugivore |
| *Rousettus madagascariensis* | 65.7 | 70.9 | 13.1 | 130.0 | 3.9 | Frugivore |
| *Rousettus obliviosus* | 45.3 | 72.5 | 8.6 |  |  | Frugivore |
| *Rousettus spinalatus* | 92.3 | 85.1 | 12.7 |  |  | Frugivore |
| *Saccolaimus flaviventris* | 45.3 | 75.0 | 8.0 |  |  | Insectivore |
| *Saccolaimus mixtus* | 38.3 | 64.5 | 9.2 |  |  | Insectivore |
| *Saccolaimus peli* | 53.4 | 89.5 | 6.7 |  |  | Insectivore |
| *Saccolaimus saccolaimus* | 43.0 | 71.2 | 8.5 |  |  | Insectivore |
| *Saccopteryx bilineata* | 8.1 | 46.0 | 3.8 |  |  | Insectivore |
| *Saccopteryx canescens* | 3.4 | 38.0 | 2.4 |  |  | Insectivore |
| *Saccopteryx gymnura* | 6.1 | 34.0 | 5.3 |  |  | Insectivore |
| *Scleronycteris ega* | 49.5 | 34.5 | 41.6 |  |  | Frugivore |
| *Scotonycteris ophiodon* | 69.4 | 76.0 | 12.0 |  |  | Frugivore |
| *Scotonycteris zenkeri* | 21.3 | 51.2 | 8.1 |  |  | Frugivore |
| *Sphaerias blanfordi* | 28.9 | 53.8 | 10.0 |  |  | Frugivore |
| *Sphaeronycteris toxophyllum* | 16.1 | 38.5 | 10.8 |  |  | Frugivore |
| *Stenoderma rufum* | 21.1 | 49.4 | 8.6 | 61.9 | 5.5 | Frugivore |
| *Sturnira aratathomasi* | 49.7 | 58.6 | 14.5 | 89.3 | 6.2 | Frugivore |
| *Sturnira bidens* | 18.1 | 41.5 | 10.5 |  |  | Frugivore |
| *Sturnira erythromos* | 15.5 | 41.7 | 8.9 |  |  | Frugivore |
| *Sturnira lilium* | 20.2 | 40.5 | 12.3 | 66.0 | 4.6 | Frugivore |
| *Sturnira ludovici* | 21.0 | 43.5 | 11.1 |  |  | Frugivore |
| *Sturnira luisi* | 12.0 | 43.0 | 6.5 |  |  | Frugivore |
| *Sturnira magna* | 27.6 | 57.5 | 8.3 |  |  | Frugivore |
| *Sturnira mordax* | 11.8 | 46.5 | 5.5 |  |  | Frugivore |
| *Sturnira nana* | 6.6 | 35.0 | 5.4 |  |  | Frugivore |
| *Sturnira thomasi* | 15.5 | 47.0 | 7.0 |  |  | Frugivore |
| *Sturnira tildae* | 24.4 | 45.5 | 11.8 |  |  | Frugivore |
| *Styloctenium wallacei* | 172.4 | 94.9 | 19.1 | 164.5 | 6.4 | Frugivore |
| *Syconycteris australis* | 17.6 | 41.9 | 10.0 | 67.4 | 3.9 | Frugivore |
| *Syconycteris carolinae* | 39.7 | 60.2 | 11.0 |  |  | Frugivore |
| *Syconycteris hobbit* | 20.1 | 47.9 | 8.8 | 68.9 | 4.2 | Frugivore |
| *Tadarida aegyptiaca* | 17.6 | 50.0 | 7.1 | 75.0 | 3.1 | Insectivore |
| *Tadarida australis* | 36.4 | 45.1 | 17.9 |  |  | Insectivore |
| *Tadarida brasiliensis* | 12.6 | 42.3 | 7.0 | 41.5 | 7.3 | Insectivore |
| *Tadarida fulminans* | 33.9 | 59.0 | 9.7 |  |  | Insectivore |
| *Tadarida lobata* | 30.4 | 59.5 | 8.6 |  |  | Insectivore |
| *Tadarida teniotis* | 28.1 | 60.0 | 7.8 | 84.0 | 4.0 | Insectivore |
| *Tadarida ventralis* | 37.4 | 64.0 | 9.1 |  |  | Insectivore |
| *Taphozous georgianus* | 30.5 | 69.2 | 6.4 |  |  | Insectivore |
| *Taphozous hamiltoni* | 39.2 | 65.0 | 9.3 |  |  | Insectivore |
| *Taphozous hildegardeae* | 29.4 | 66.5 | 6.6 |  |  | Insectivore |
| *Taphozous hilli* | 22.0 | 67.5 | 4.8 |  |  | Insectivore |
| *Taphozous kapalgensis* | 26.5 | 60.3 | 7.3 |  |  | Insectivore |
| *Taphozous longimanus* | 25.1 | 58.5 | 7.3 |  |  | Insectivore |
| *Taphozous mauritianus* | 28.0 | 61.7 | 7.3 | 85.0 | 3.9 | Insectivore |
| *Taphozous melanopogon* | 26.0 | 63.7 | 6.4 |  |  | Insectivore |
| *Taphozous nudiventris* | 32.5 | 86.0 | 4.4 |  |  | Insectivore |
| *Taphozous nudiventris* | 8.9 | 48.0 | 3.9 |  |  | Insectivore |
| *Taphozous perforatus* | 24.4 | 62.0 | 6.4 | 72.5 | 4.6 | Insectivore |
| *Taphozous theobaldi* | 36.8 | 71.0 | 7.3 |  |  | Insectivore |
| *Taphozous troughtoni* | 53.5 | 72.5 | 10.2 |  |  | Insectivore |
| *Thoopterus nigrescens* | 66.1 | 72.3 | 12.6 | 104.5 | 6.1 | Frugivore |
| *Thyroptera discifera* | 3.1 | 33.5 | 2.8 |  |  | Insectivore |
| *Thyroptera tricolor* | 4.5 | 35.5 | 3.6 |  |  | Insectivore |
| *Tomopeas ravus* | 5.6 | 33.0 | 5.2 |  |  | Insectivore |
| *Tonatia bidens* | 27.7 | 58.1 | 8.2 |  |  | Omnivore |
| *Tonatia brasiliensis* | 9.8 | 36.5 | 7.3 |  |  | Omnivore |
| *Tonatia carrikeri* | 22.4 | 46.5 | 10.3 |  |  | Omnivore |
| *Tonatia evotis* | 20.6 | 50.0 | 8.2 |  |  | Omnivore |
| *Tonatia schulzi* | 18.0 | 42.6 | 9.9 |  |  | Omnivore |
| *Tonatia silvicola* | 32.3 | 54.5 | 10.9 |  |  | Omnivore |
| *Trachpos cirrhosus* | 36.9 | 58.5 | 10.8 |  |  | Omnivore |
| *Triaenops furculus* | 5.6 | 44.0 | 2.9 | 67.5 | 1.2 | Insectivore |
| *Triaenops persicus* | 13.2 | 53.0 | 4.7 |  |  | Insectivore |
| *Tylonycteris pachypus* | 4.1 | 26.2 | 6.0 |  |  | Insectivore |
| *Tylonycteris robustula* | 8.0 | 28.0 | 10.2 |  |  | Insectivore |
| *Uroderma bilobatum* | 16.3 | 42.0 | 9.2 |  |  | Frugivore |
| *Uroderma magnirostrum* | 17.3 | 41.5 | 10.0 |  |  | Frugivore |
| *Vampyressa bidens* | 11.9 | 35.6 | 9.4 |  |  | Frugivore |
| *Vampyressa brocki* | 48.0 | 33.5 | 42.8 |  |  | Frugivore |
| *Vampyressa melissa* | 16.6 | 38.0 | 11.5 |  |  | Frugivore |
| *Vampyressa nymphaea* | 69.0 | 36.5 | 51.8 |  |  | Frugivore |
| *Vampyressa pusilla* | 8.8 | 30.5 | 9.4 | 53.0 | 3.1 | Frugivore |
| *Vampyrodes caraccioli* | 35.9 | 52.1 | 13.2 |  |  | Frugivore |
| *Vampyrum spectrum* | 171.6 | 68.8 | 36.3 |  |  | Omnivore |
| *Vespadelus darlingtoni* | 6.1 | 34.0 | 5.2 |  |  | Insectivore |
| *Vespertilio murinus* | 15.4 | 44.8 | 7.7 |  |  | Insectivore |
| *Vespertilio superans* | 24.3 | 48.5 | 10.3 |  |  | Insectivore |

Appendix S2. Scatter diagrams of full body and forearm length data for bats. Full body length and forearm length data are shown in millimeters and the linear regression curves and correlation coefficients (R^2^) are shown.


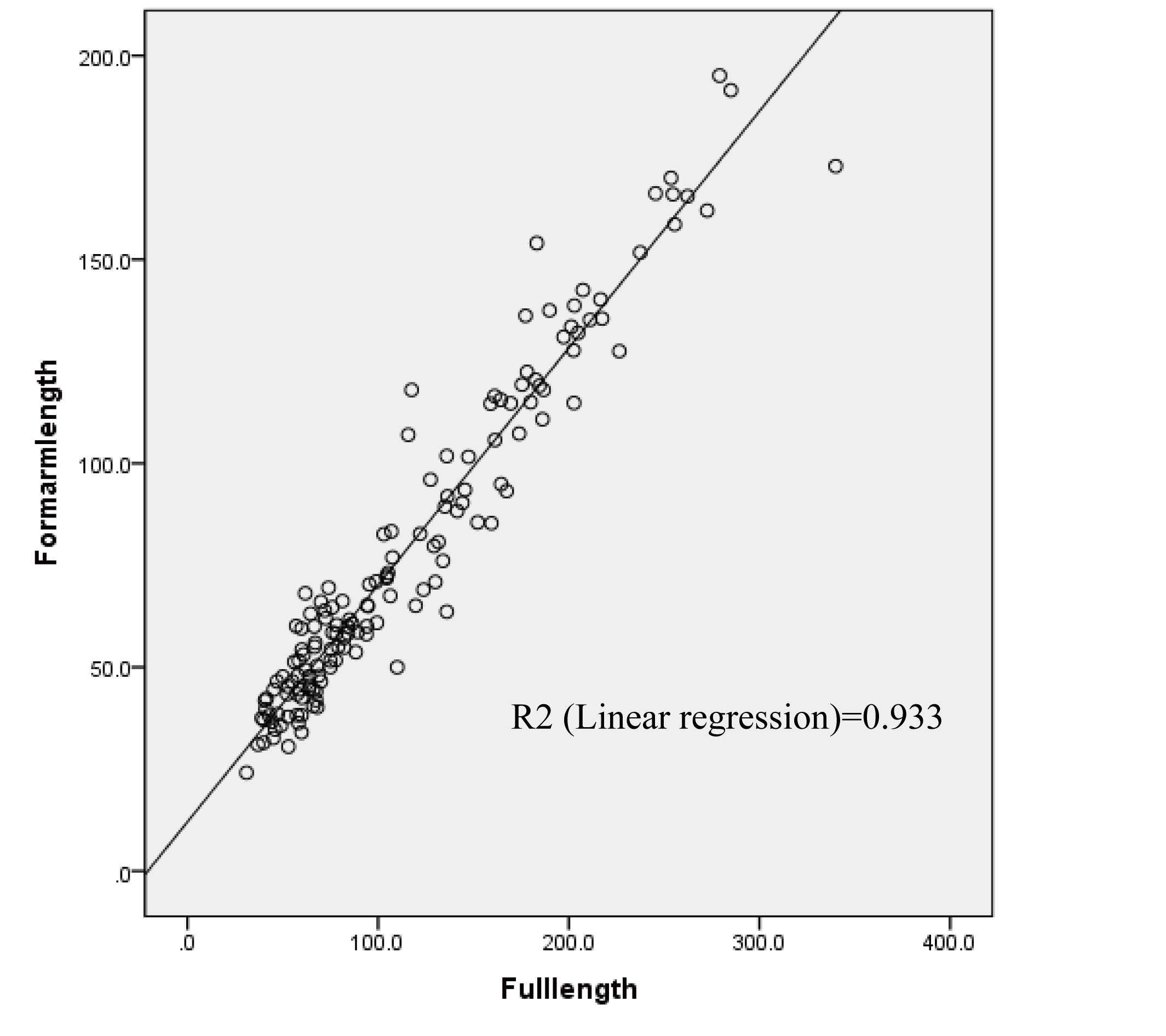


Appendix S3. Scatter diagram of forearm mass indexes (FMI) and body weights for New World fruit-eating (NWFBs) and Old World fruit-eating (OWFBs) bats. NWFBs and OWFBs are shown in red and green triangles, respectively.


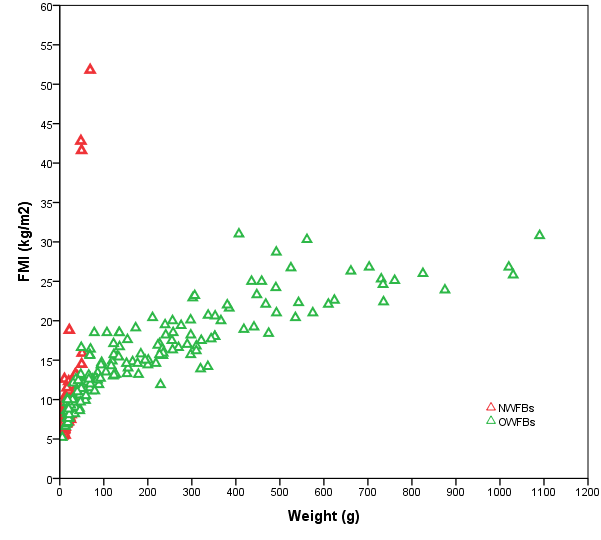


Appendix S4. FMI, BMI, dietary, and blood glucose level data of the studied bats. Data are presented in descending order of BMI values. Data for *Scotophilus heathi*^1^, *Epomophorus wahlbergi*^2^, *Pteropus vampyrus*^3^, *P. hypomelanus*^3^, *Rousettus aegyptiacus*^3^, *Taphozous nudiventris*^4^, and *Nyctus noctula*^5^ were retrieved from published papers, with means of the data, and their upper and lower limits, used for calculating BMI and FMI. Data for the remaining bats (including *Cynopterus sphinx*, *Myotis daubentonii*, *M. ricketti*, *Rhinolophus affinis*, *R. sinicus*, *R. ferrumequinum*, *Rousettus leschenaulti*, *Hipposideros armiger*, and *H. pratti*) were obtained in this work.

| Species name | Body weight | Forearm length | FMI | Full length | BMI | Blood glucose | Diet |
| --- | --- | --- | --- | --- | --- | --- | --- |
|  | g | mm | kg/m^2^ | mm | kg/m^2^ | mmol/L |  |
| *Cynopterus sphinx* | 50.5 | 70.5 | 10.2 | 96.5 | 5.4 | 8.3 | Frugivore |
| *Epomophorus wahlbergi* | 93.6 | 80.7 | 14.4 | 131.7 | 5.4 | 6.5 | Frugivore |
| *Hipposideros armiger* | 54.5 | 93.0 | 6.3 | 78.0 | 9.0 | 4.2 | Insectivore |
| *Hipposideros pratti* | 57.4 | 87.7 | 7.5 | 78.1 | 9.4 | 5.2 | Insectivore |
| *Myotis daubentonii* | 5.2 | 34.9 | 4.3 | 39.6 | 3.3 | 8.9 | Insectivore |
| *Myotis ricketti* | 17.8 | 55.6 | 5.8 | 56.6 | 5.6 | 7.1 | Insectivore |
| *Nyctus noctula* | 20.5 | 49.0 | 8.5 | 72.0 | 4.0 | 6.4 | Insectivore |
| *Pteropus hypomelanus* | 516.0 | 199.5 | 13.0 | 305.0 | 5.5 | 5.0 | Frugivore |
| *Pteropus vampyrus* | 680.0 | 200.0 | 17.0 | 220.0 | 14.0 | 4.9 | Frugivore |
| *Rhinolophus affinis* | 12.5 | 51.3 | 4.7 | 51.4 | 4.7 | 9.5 | Insectivore |
| *Rhinolophus ferrumequinum* | 22.3 | 59.5 | 6.3 | 52.5 | 8.1 | 5.2 | Insectivore |
| *Rhinolophus sinicus* | 12.2 | 48.1 | 5.3 | 46.5 | 5.6 | 8.0 | Insectivore |
| *Rousettus aegyptiacus* | 136.0 | 94.0 | 15.4 | 150.0 | 6.0 | 6.0 | Frugivore |
| *Rousettus leschenaulti* | 91.1 | 77.2 | 15.3 | 126.2 | 5.7 | 5.2 | Frugivore |
| *Scotophilus heathi* | 36.1 | 61.5 | 9.5 | 85.0 | 5.0 | 4.5 | Insectivore |
| *Taphozous nudiventris* | 32.5 | 76.2 | 5.6 | 48.0 | 14.1 | 4.9 | Insectivore |

Reference

1. Srivastava, R. K. & Krishna, A. Melatonin modulates glucose homeostasis during winter dormancy in a vespertilionid bat, *Scotophilus heathi*. *Comp Biochem Physiol A Mol Integr Physiol* **155**, 392-400, doi:10.1016/j.cbpa.2009.12.006 (2010).

2. Downs, C. T., Mqokeli, B. & Singh, P. Sugar assimilation and digestive efficiency in Wahlberg's epauletted fruit bat (*Epomophorus wahlbergi*). *Comp Biochem Physiol A Mol Integr Physiol* **161**, 344-348, doi:10.1016/j.cbpa.2011.12.003 (2012).

3. Widmaier, E. P. & Kunz, T. H. Basal, diurnal, and stress-induced levels of glucose and glucocorticoids in captive bats. *J Exp Zool* **265**, 533-540, doi:10.1002/jez.1402650509 (1993).

4. Desai, I., Pandya, H., Pratyush, P. & Suresh, B. Some observations on the population of naked-rumped tomb bat (*Taphozous nudiventris* Cretzschmar) at the Maharaja Sayajirao University of Baroda campus, Gujarat. *Cibtech J Zool* **1**, 27-35 (2012).

5. Li, D. Season changes of lactate dehydrogenase isoenzyme and blood glucose of *Nyctus noctula* and *Rana nigromaculata* (in Chinese). *Acta Physiol Sin* **46**, 267-272 (1994).
